# Fabrication of BaZr_0.1_Ce_0.7_Y_0.1_Yb_0.1_O_3‐δ_ Skeleton Structure for the Development of High‐Performance Nanocomposite Cathodes for Protonic Ceramic Fuel Cells by Nitrate Impregnation

**DOI:** 10.1002/smll.74677

**Published:** 2026-07-25

**Authors:** Jialin Li, Zhongwei Yue, Zhishan Li, Yu Shen, Fang Wang, San Ping Jiang

**Affiliations:** ^1^ Foshan Xianhu Laboratory Foshan People's Republic of China; ^2^ School of Materials Science and Engineering Changchun University of Science and Technology Changchun People's Republic of China; ^3^ Zhongshan Institute of Changchun University of Science and Technology Zhongshan People's Republic of China; ^4^ WA School of Mines: Minerals, Energy & Chemical Engineering Curtin University Perth WA Australia

**Keywords:** BaZr_0.1_Ce_0.7_Y_0.1_Yb_0.1_O_3‐δ_ electrolyte skeleton, impregnation, nanocomposite cathode, performance and stability, protonic ceramic fuel cells

## Abstract

A stable and interconnected skeleton structure is critical for the fabrication of high‐performance nanocomposite cathodes by impregnation for protonic ceramic cells (PCCs). This work systematically synthesizes an interconnected BaZr_0.1_Ce_0.7_Y_0.1_Yb_0.1_O_3‐δ_ (BZCYYb1711) electrolyte skeleton structure on a dense BZCYYb1711 electrolyte and introduces three catalytic active materials by nitrate impregnation: the electronic conducting La_0.8_Sr_0.2_MnO_3‐δ_ (LSM), mixed ionic and electronic conducting La_0.6_Sr_0.4_Co_0.2_Fe_0.8_O_3‐δ_ (LSCF) and triple conducting PrBa_0.5_Sr_0.5_Co_1.5_Fe_0.5_O_5+δ_ (PBSCF). The results indicate that skeleton formation and interconnectivity are strongly associated with sintering temperature, most likely governed by the thermodynamics of densification of Ba‐containing electrolyte ceramics. The electrode performance strongly correlates with the activity of the impregnated oxides. The PBSCF impregnated BZCYYb1711 (PBSCF‐1711) electrode exhibits the best performance with a low R_p_ value of 0.06 Ω cm^2^ at 700°C in wet air. The fuel cell with PBSCF‐1711 cathode shows a maximum power density of 486 mW cm^−2^ at 700°C and demonstrates excellent durability, operating stably for over 700 h in fuel cell (FC) mode and through cyclic fuel cell/electrolysis cell operation at 0.5 A cm^−2^ in H_2_/wet air for 100 h. This study shows the potential for the development of high‐performance air electrodes on a stable and interconnected porous skeleton structure based on nitrate impregnation for PCCs.

## Graphical abstract

A homologous BZCYYb1711 cathode skeleton was constructed using the same material as the electrolyte, and active components were introduced into the skeleton via nitrate impregnation to form a nanocomposite cathode. This strategy effectively alleviated the thermal expansion coefficient (TEC) mismatches between the cathode and electrolyte. During long‐term operation, continuous microstructural evolution of the composite cathode exposed more active sites, leading to steadily improved electrochemical performance and stable operation for over 700 h.

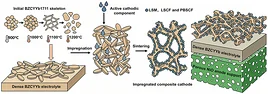

## 1 Introduction

Protonic ceramic fuel cells (PCFCs) offer advantages such as high energy conversion efficiency, fuel flexibility and minimal greenhouse gas emissions, similar to the high temperature solid oxide fuel cells (SOFCs) [1, 2, 3, 4], but different to the high operating temperature (800–1000°C) typically associated with SOFCs [5, 6], PCFCs can operate at low to intermediate temperatures of 500–700°C due to the fast and facile proton migration properties of proton conducting perovskite electrolyte materials [6, 7]. A key advantage of PCFCs is the generation of water on cathode, which prevents fuel dilution and enhances energy conversion efficiency [8]. Nevertheless, one of the limiting factors in the high performance of PCFCs is the sluggish oxygen reduction reaction (ORR) at the cathode [9]. The cathode reaction involves complex and slow charge and mass transfer processes among electrolyte, cathode, and gas phase, resulting in an electrode polarization resistance, R_p_, that is significantly larger than that of the hydrogen oxidation reaction at the anode and the ohmic resistance, R_o_, of the thin electrolyte layer [10].

In general, cathode materials can be primarily categorized into two groups: single‐phase and multi‐phase materials. Single‐phase materials can be further classified based on the type of charge carriers they conduct: metal**/**hole‐conducting oxides and mixed conducting oxides. Representative materials of metal**/**hole‐conducting oxides include metallic platinum (Pt), which conducts only electrons, and perovskite oxides such as La_0.8_Sr_0.2_MnO_3‐δ_ (LSM), which conducts almost exclusively electron holes [11, 12]. However, these materials generally exhibit high R_p_ because their reactions are confined to the triple‐phase boundary (TPB) where electrolyte, electrode and reaction gas meet. Unlike SOFCs, water generated at the PCFC cathode may occupy active sites for the oxygen adsorption, leading to an increase in the electrode polarization resistance with rising water vapor pressure ($p_{H_{2}O}$) [12]. The mixed conducting oxides build upon the oxygen‐ion and electron hole‐conducting cathodes commonly used in SOFCs by incorporating proton conduction properties, forming triple conducting oxides (TCOs) [13]. Common TCOs often adopt perovskite, double perovskite and Ruddlesden‐Popper structures, such as BaCo_0.4_Fe_0.4_Zr_0.1_Y_0.1_O_3‐δ_ (BCFZY), PrBa_0.5_Sr_0.5_Co_1.5_Fe_0.5_O_5+_ *_δ_* (PBSCF) and Pr_2_NiO_4+_ *_δ_* (PNO) [14, 15, 16, 17].

Multi‐phase materials can be categorized into composite systems such as perovskite oxides/perovskite oxides, Ruddlesden‐Popper oxides/perovskite oxides, perovskite oxides/binary metal oxides and perovskite oxides/metals [13]. The methods of constructing multi‐phase cathode composites mainly include mechanical mixing, self‐assembly, impregnation**/**infiltration and exsolution [13]. Among them, mechanical mixing is simple and easy to use. Kong et al. employed mechanical mixing to prepare CuCo_2_O_4_‐Er_0.4_Bi_1.6_O_3‐δ_ composite cathode and reported a R_p_ of 0.07 Ω cm^2^ in air at 800°C [18]. However, the interface formed by physical mixing has poor stability under long‐term high‐temperature operation and is prone to phase separation [19]. The composite cathode obtained by self‐assembly method contains more catalytically active nanoparticles as reactive sites [20]. However, this method is highly sensitive to parameters such as precursor stoichiometric ratio, sintering temperature, time, and atmosphere, resulting in relatively poor reproducibility [21]. Exsolution exhibits excellent stability due to its strong anchoring effect with the electrode framework, which is beneficial for long‐term operation, but it is mostly used in anode under reducing conditions [22]. Li et al. doped cobalt (Co) into BaCe_0.7_Zr_0.2_Y_0.1_O_3‐δ_ (BCZY172), and Co nanoparticles precipitated on BCZY skeleton surface through reduction treatment, forming R‐BCZY‐Co_x_ (x = 0.05, 0.1, 0.15) composite electrodes. The precipitated Co nanoparticles provide abundant reaction sites, resulting in an R_p_ of 0.6 Ω cm^2^ for the symmetric cell with R‐BCZY‐Co_0.1_ cathode, significantly lower than 3.5 Ω cm^2^ obtained on the cell with BCZY cathode [23].

Impregnation is one of the most effective methods to form multi‐phase composite systems with high catalytic activity and stability [14, 24]. For instance, Wei et al. impregnated La_2_NiO_4+δ_ (LNO) onto La_0.6_Sr_0.4_Co_0.2_Fe_0.8_O_3‐δ_ (LSCF) scaffold, reducing R_p_ of cathode and improving electrochemical performance of PCFCs [25]. Xi et al. impregnated nano‐scale Sm_0.5_Sr_0.5_CoO_3‐δ_ (SSC) particles on PrBaCo_2_O_5+δ_ (PBC) cathode surface, the total resistance decreased from 0.8 to 0.5 Ω cm^2^ under open circuit voltage at 700°C [26]. Zhou et al. developed Ni‐YSZ metal‐supported SOFCs with impregnated SrFe_0.75_Mo_0.25_O_3_ (SFMO‐YSZ) cathodes, achieving a R_p_ of 0.06 Ω cm^2^ at 800°C in air and a maximum power densities of 438 mW cm^−2^ at 800°C [27]. Yao et al. reported that Pr_2_Ni_0.5_Co_0.5_O_4‐δ_ impregnated La_0.6_Sr_0.4_Co_0.2_Fe_0.8_O_3‐δ_ (PNC‐LSCF) cathodes yielded a maximum power density of 1857 mW cm^−2^ at 700°C [28]. However, such Co‐containing active materials exhibit high thermal expansion coefficients (TECs) of 13.6–26.1 × 10^−6^ K^−1^, much higher than 9.7 × 10^−6^ K^−1^ typically associated with proton conducting electrolyte materials [29, 30, 31]. The TECs mismatch between electrolyte and electrode would lead to degradation, delamination or shedding at operating temperature (400–700°C), which hinders the performance stability of PCFCs [32].

One effective approach is to form thermally matching skeleton on the electrolyte substrates, followed by impregnation of active components. In SOFCs, the sintering temperature used to fabricate porous skeleton is typically in the range of 1200 to 1400°C [33, 34, 35, 36]. Jiang et al. formed Ni skeleton on yttria stabilized zirconia (YSZ) electrolyte surface by sintering at 1400°C and impregnated 8.5 wt.% gadolinium‐doped ceria (GDC) into Ni skeleton, forming nanocomposite Ni/GDC electrodes, achieving electrode polarization resistance of 0.71 Ω cm^2^ at 800°C [35]. Okabe et al. deposited GDC‐YSZ composite anode onto YSZ electrolyte by screen‐printing and subsequently sintered the electrodes at 1200°C [37]. In PCFCs, the temperatures for forming a porous skeleton are generally lower in the range of 1000 to 1200°C. Liu et al. prepared a Sm_0.5_Sr_0.5_CoO_3_‐BaZr_0.1_Ce_0.7_Y_0.2_O_3_ (BZCY172) composite cathode (weight ratio = 7:3) using a suspension spray process, followed by sintering at 1000°C for 3 h [38]. Chen et al. mixed LSCF, BZCYYb1711 and starch (mass ratio 6:4:2) with ethyl cellulose‐terpineol binder to prepare cathode slurry. The slurry was subsequently coated onto BZCYYb1711 electrolyte layer and sintered at 1100°C for 2 h [39]. These are cases of fabricating skeleton by mixing pure proton conductors and TCOs, whereas reports on preparing skeleton using a single proton conductor are relatively scarce. Li et al. screen‐printed pure BZCY172 slurry onto BZCY electrolyte and sintered the sample at 1100°C for 5 h to form a porous skeleton [23]. Li et al. also prepared BZCY172 slurry for screen‐printing BZCY electrolyte, then the sample was sintered at 1200°C for 3 h [40]. Currently, there are no reports on pure proton conductor skeleton impregnated with active components to form PCFC composite cathodes. The optimal sintering temperature for skeleton remains undetermined, and the effects of impregnation on skeleton morphology, as well as the mechanisms of different impregnated components for cathode enhancement remain unclear.

To address the problems mentioned above, in this work we reported the development of multi‐phase composite cathode materials by forming a BaZr_0.1_Ce_0.7_Y_0.1_Yb_0.1_O_3‐δ_ (BZCYYb1711) skeleton onto the BZCYYb1711 electrolyte layer and introducing the active components by nitrate impregnation method to enhance the electrochemical activity as well as to mitigate TECs mismatch between the composite cathode and electrolyte. The morphology of BZCYYb1711 skeleton was compared after sintering at different temperatures (900–1200°C) for 2 h. The best temperature for the formation of BZCYYb1711 skeleton on BZCYYb1711 electrolyte was found to be 1100°C. Three common active components, electronic conducting La_0.8_Sr_0.2_MnO_3‐δ_ (LSM), mixed ionic and electronic conducting (MIEC) La_0.6_Sr_0.4_Co_0.2_Fe_0.8_O_3‐δ_ (LSCF) and triple conducting oxide (TCO) PrBa_0.5_Sr_0.5_Co_1.5_Fe_0.5_O_5+δ_ (PBSCF) were selected and introduced into the BZCYYb1711 skeleton by impregnation. The results showed that the impregnated component forms a single perovskite phase after sintering at 1100°C for 2 h, and is uniformly distributed on the skeleton as nanoparticles. Impregnation effectively reduced the electrode polarization resistance (R_p_) and ohmic resistance (R_o_) of the cells. In three‐electrode cells, after 100 h long‐term stability test under electrolysis cell (EC) mode, the voltage required to maintain a constant current decreased and the electrochemical performance of cells improved. The single cell with impregnated PBSCF‐1711 air electrode exhibits excellent stability during the 100 h cycling test when switching between fuel cell (FC) and electrolysis cell (EC) modes and operates stably for 734 h in FC mode.

## 2 Results and Discussion

### 2.1 BZCYYb1711 Skeleton Formation

**FIGURE 1:**
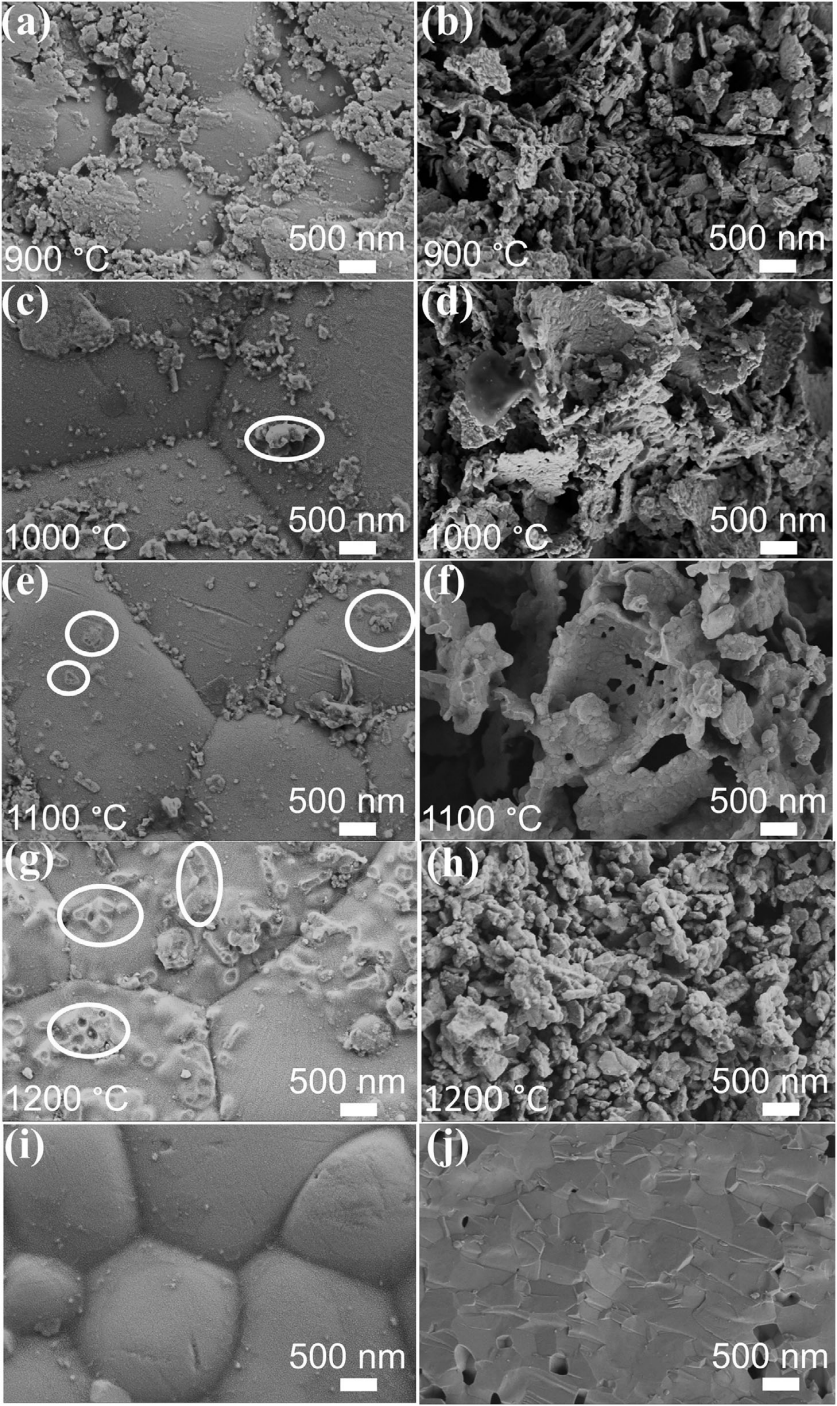
Microstructure of surface and cross‐section of BZCYYb1711 electrolyte with BZCYYb1711 skeleton sintered at (a,b) 900°C, (c,d) 1000°C, (e,f) 1100°C, and (g,h) 1200°C for 2 h. Microstructure of the surface and cross‐section of pristine BZCYYb1711 electrolyte are shown in (i,j). The BZCYYb1711 skeleton was removed by sticking paper method to observe the surface of BZCYYb1711 electrolyte.

The BZCYYb1711 skeleton formation temperature and the interface between the BZCYYb1711 skeleton and BZCYYb1711 electrolyte sintered at 900, 1000, 1100, and 1200°C were first studied and the results are shown in Figure 1. The surface of pristine BZCYYb1711 electrolyte without BZCYYb1711 skeleton is clean and is characterized with the formation of grains with size in the range of 1.05 to 5.13 µm (the average is ∼2.8 µm) (Figure 1i and Figure S2a). The BZCYYb1711 electrolyte shows dense microstructure with few isolated pores, indicating the densification of BZCYYb1711 electrolyte after sintering at 1450°C. For the BZCYYb1711 skeleton sintered at 900°C, there exists BZCYYb1711 particles on the BZCYYb1711 electrolyte surface after removal of the skeleton by the sticking paper method (Figure 1a). The cross‐section of the skeleton is characterized by the formation of small round‐shaped particles as well as plate‐like BZCYYb1711 particles (Figure 1b) and the diameter of the particles is in the range of 0.16 to 0.71 µm (the average is ∼ 0.36 µm) (Figure S1a). There is no intimate bonding between the small plate‐like particles (Figure 1b). The particles left on the electrolyte surface can also be easily removed, indicating no strong bonding or interface formation between the BZCYYb1711 skeleton and BZCYYb1711 electrolyte. As the sintering temperature increased to 1000°C, there is growth of the BZCYYb1711 plate‐like particles with average diameter of 0.70 µm with the substantial reduction of round‐shaped small particles (Figure 1d and Figure S1b). The plate‐like particles are partially interconnected and there are also significant number of isolated plate‐like particles, indicating the formation of an incomplete and weak skeleton structure (Figure 1d). This indicates the joining of small round‐shaped particle into large plate‐like particle with the increase of the sintering temperature. There is an observation of BZCYYb1711 fragments strongly adhered to the BZCYYb1711 electrolyte substrate (circles, Figure 1c), indicating the initial stage of the interface formation between the skeleton and the electrolyte.

**FIGURE 2:**
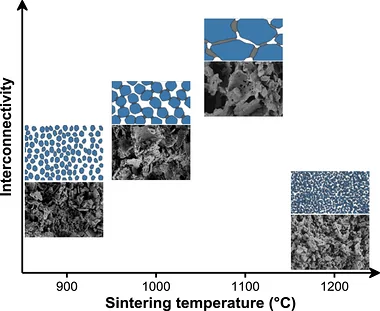
Scheme of the interconnectivity of BZCYYb1711 skeleton and interface formation as a function of sintering temperatures.

With the increase of sintering temperature to 1100°C, plate‐like particles are interconnected with no isolated particles with clean surface (Figure 1f), indicating the formation of BZCYYb1711 skeleton structure with a fully interconnected network. The diameter of the plate‐like particles also grows to as large as 2.09 µm (Figure S1c). Such interconnected BZCYYb1711 skeleton structure is very similar to the reported BaCe_0.7_Zr_0.2_Y_0.1_O_3‐δ_ (BCZY172) skeleton after sintering at 1100°C for 2 h [23, 40]. The formation of skeleton network is also indicated by the strongly adhered BZCYYb1711 particles and contact marks observed on the BZCYYb1711 electrolyte (circles, Figure 1e). The formation of contact marks is an indication of the interface formation between the BZCYYb1711 skeleton and BZCYYb1711 electrolyte, similar to that reported for the interface formation between LSM/YSZ and LSCF/YSZ in the case of SOFCs [41, 42]. When the sintering temperature was further increased to 1200°C, the plate‐like particles disappear, forming small particles and plates with size in the range of 0.09–0.96 µm (Figure 1h and Figure S1d). The small particles and plates form large agglomerated particles as large as 1–1.2 µm, but are not interconnected, indicating the collapse and disintegration of the interconnected plate‐like skeleton network structure. On the BZCYYb1711 electrolyte surface, there is significant formation of large number of contact marks and the surface of the BZCYYb1711 electrolyte grains becomes rough as compared to the pristine BZCYYb1711 electrolyte surface (Figure 1g,i), showing the coarsening of the interfacial contact between BZCYYb1711 skeleton and BZCYYb1711 electrolyte. The change from the plate‐like interconnected structure to round‐shaped particles with the increase of the sintering temperature from 1100°C to 1200°C is most likely due to the reduction of the surface energy of the particles driven by the thermodynamics of densification during the ceramic sintering process. The results show that there is a close relationship between the interconnectivity of the skeleton network, average size of skeleton and sintering temperature, as shown schematically in Figure 2. The average size of BZCYYb1711 skeleton increased from 0.36 to 1.89 µm as the sintering temperature rising from 900 to 1100°C with enhanced interconnectivity (Figure 2 and Figure S1a–c). However, when the sintering temperature further increased to 1200°C, the skeleton structure collapsed, reducing the average size to 0.26 µm and the interconnected morphology nearly disappeared (Figure 2 and Figure S1d).

During the low sintering temperature region (900–1100°C), the small round‐shaped as well as plate‐like particles are joining together to form large plate‐like particles. With the increase of the sintering temperature (1200°C), the large and interconnected plate‐like particles collapse, forming small round‐shaped particles with significant agglomeration, driven by the thermodynamic of densification during the ceramic sintering processes. The interconnectivity is associated with the average size of particles. Cross‐sectional SEM images of symmetric cells sintered at 900–1200°C further confirm that the BZCYYb1711 skeleton remains closely bonded to the BZCYYb1711 electrolyte at all tested sintering temperatures, with no obvious interfacial separation or cracking (Figure S8). Based on SEM observations and interfacial contact features, the best condition for the formation of interconnected and stable BZCYYb1711 skeleton structure on the BZCYYb1711 electrolyte with excellent interface is the sintering at 1100°C for 2 h in air. Thus, in following sections, the BZCYYb1711 skeleton was all sintered at 1100°C for 2 h in air. In addition, the BZCYYb1711 paste used for screen printing was sintered at 1100°C, followed by Brunauer‐Emmett‐Teller (BET) analysis (Figure S11). The BET surface area was 76 m^2^ g^−1^.

### 2.2 Phase and Microstructure of the Impregnated Nanocomposite Electrodes

**FIGURE 3:**
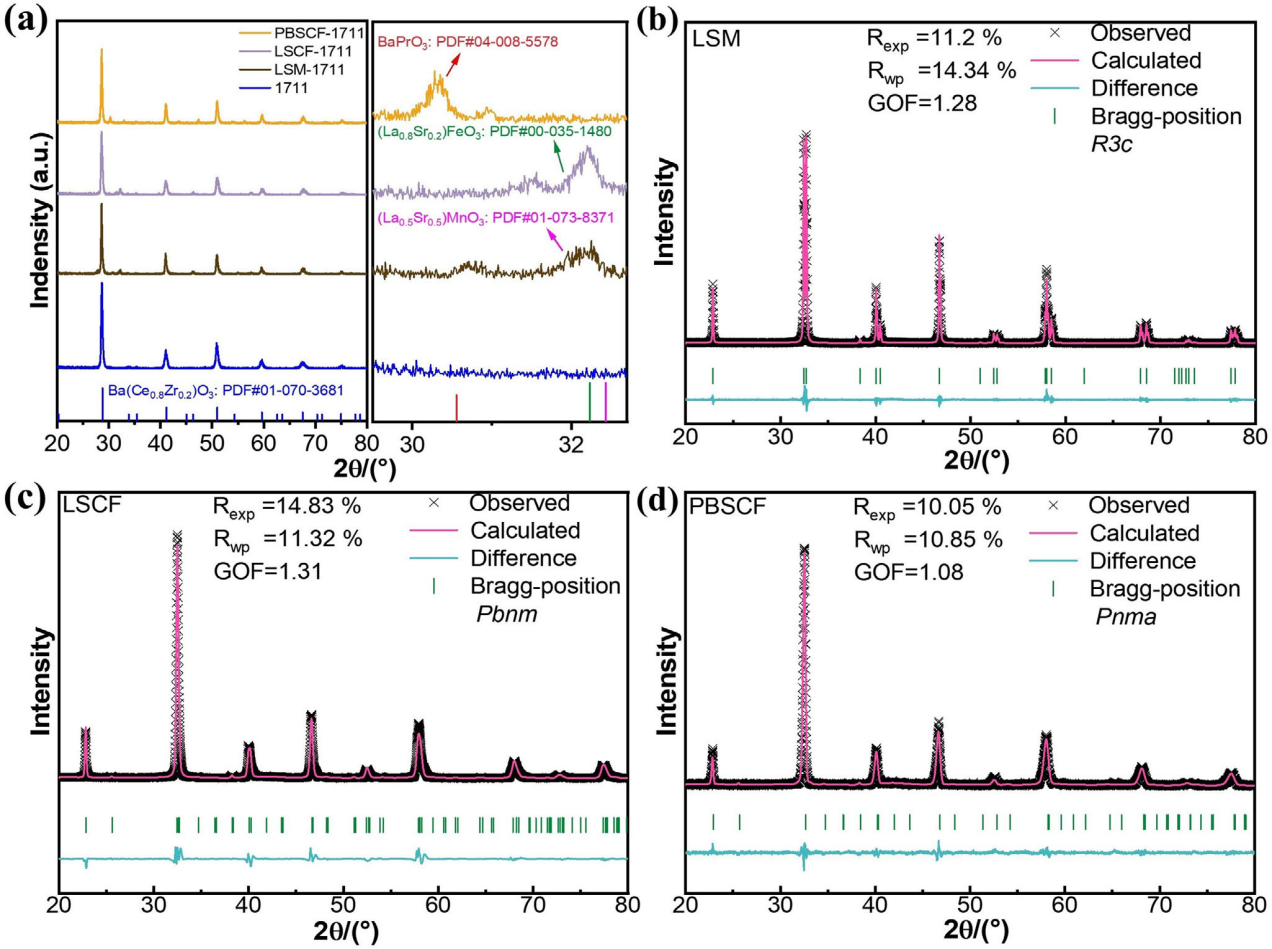
(a) XRD patterns on the surface of 1711 (initial BZCYYb1711 skeleton), LSM‐1711 (LSM impregnated BZCYYb1711 skeleton), LSCF‐1711 (LSCF impregnated BZCYYb1711 skeleton) and PBSCF‐1711 (PBSCF impregnated BZCYYb1711 skeleton) pellets. Rietveld refinement profiles of the (b) LSM, (c) LSCF and (d) PBSCF powders obtained by sintering the impregnation solution at 1100°C for 2 h.

Figure 3a presents the XRD patterns of BZCYYb1711 pellets with BZCYYb1711 skeleton surfaces before and after impregnation with LSM, LSCF, and PBSCF. Due to the thin impregnation layer and low loading density (5 mg cm^−2^), the primary signal in the XRD pattern corresponds to BZCYYb1711. For nanocomposite LSM‐1711, LSCF‐1711, and PBSCF‐1711, the formation or perovskite La_0.5_Mn_0.5_O_3_, (La_0.8_Sr_0.2_)FeO_3_ and BaPrO_3_ phases were determined even though the intensities of the phase were very low due to the low content and thin impregnation layer. This observation indicates that the impregnated components were uniformly deposited on the BZCYYb1711 skeleton, and the weak reflections are consistent with the corresponding target phases (LSM, LSCF, and PBSCF). Although the impregnation loading was low, weak peaks of the impregnated active components were still observed in the BZCYYb1711 skeleton after impregnation. To minimize the interference from the BZCYYb1711 skeleton diffraction signals and further confirm the crystal phases of the impregnated components, a small amount of the impregnation solution was separately calcined at 1100°C for 2 h. The Rietveld refinement result of the LSM, LSCF, and PBSCF powders obtained by heat‐treatment of the nitrate impregnation solution show the formation of single perovskite phases (Figure 3b–d and Table S1). For the LSM powder, a trigonal perovskite structure (space group R3c) was calculated with lattice parameters of a = b = 5.516 Å, c = 13.356 Å [43]. The LSCF and PBSCF powders form stable orthorhombic perovskite structures corresponding to space groups Pbnm and Pnma, respectively [44, 45]. The XRD analysis from powders obtained by sintering the solution serves only as supporting evidence and does not directly represent the actual phase composition on the cathode surface. The above results indicate that the impregnated components were successfully deposited as nanoparticles in the forms of single perovskite phases onto BZCYYb1711 skeleton by nitrate impregnation. The detection of minor La_0.5_Mn_0.5_O_3_, (La_0.8_Sr_0.2_)FeO_3_ and BaPrO_3_ phases also indicates the formation of multi‐phase nanocomposite.

**FIGURE 4:**
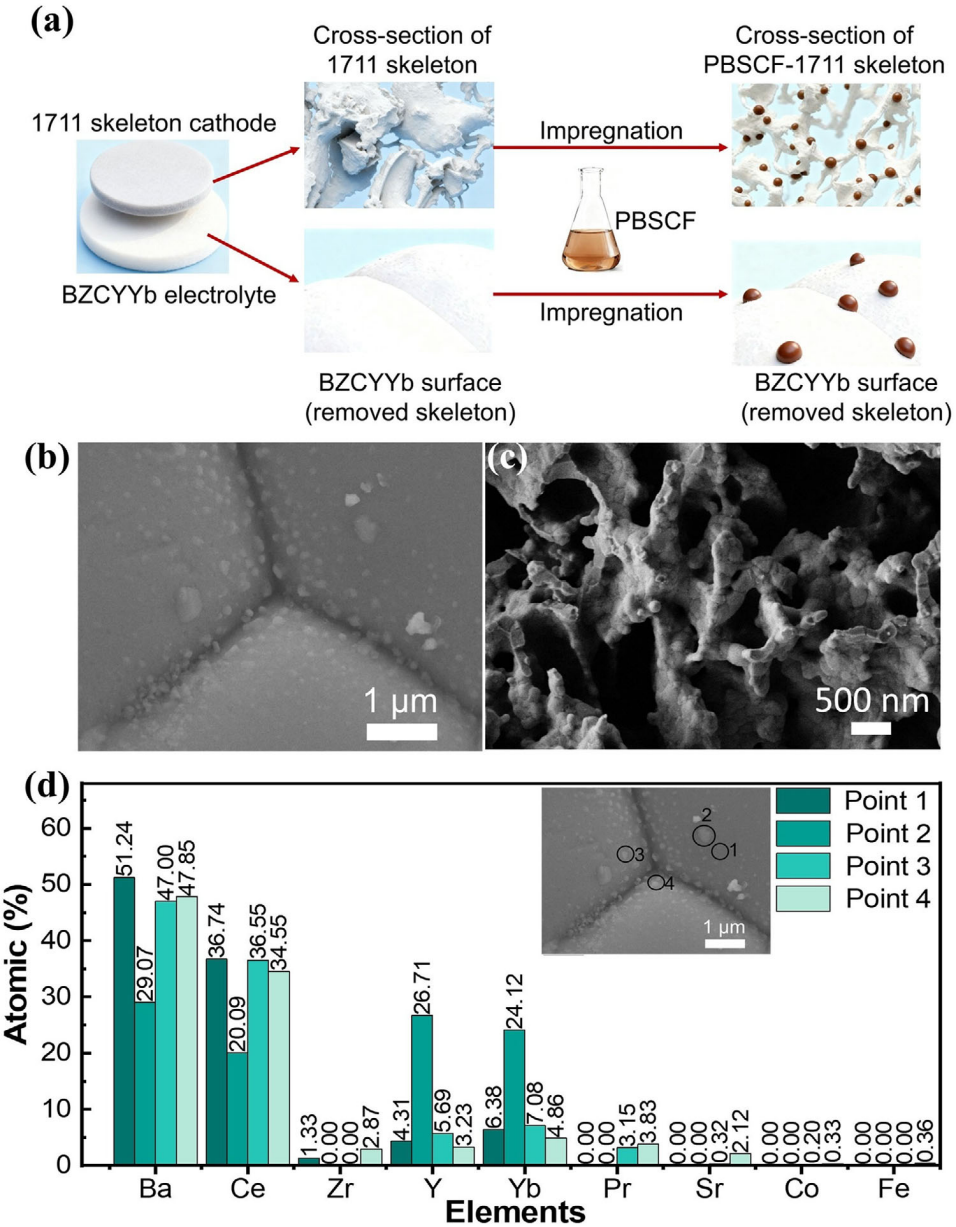
(a) Schematic diagram of PBSCF impregnation process on the BZCYYb1711 surface (after removal of the skeleton) and cross‐section. SEM pictures of (b) surface of PBSCF‐1711 after removal of the skeleton and (c) cross‐section of PBSCF‐1711 skeleton. The corresponding EDX point scan analysis of PBSCF‐1711 surface after removal of the skeleton are shown in (d).

The interface and microstructure of the nanocomposite electrodes were further investigated on the PBSCF‐1711 electrode and the results are shown in Figure 4. In the figure, the structural changes on the surface of BZCYYb1711 electrolyte and cross‐section of skeleton during the formation of PBSCF‐1711 is schematically shown in Figure 4a. After the impregnation of BZCYYb1711 skeleton with PBSCF nitrate solution, nanoparticle formation occurs on the grain surface as well as at the grain boundaries of the BZCYYb1711 electrolyte (Figure 4b). The cross‐section of the skeleton indicates the formation of interconnected three‐dimensional network with a thin impregnated layer on the surface of plate‐like particles (Figure 4c).

To investigate the impact of impregnation on the contact interface between the electrolyte and the skeleton, the elemental analysis was conducted on surfaces of pristine BZCYYb1711 (Figure S3a), BZCYYb‐1711 and PBSCF‐1711 after removal of the skeleton (Figure S3b,c), cross‐section of PBSCF‐1711 (Figure S4). For PBSCF‐1711 skeleton, there exists enrichment region of Sr and Co elements (Figure S4). For clean BZCYYb1711 electrolyte samples before and after screen printing and sintering skeleton treatment, the results indicate that only Y and Yb were segregated on surface, while barium, cerium and zirconium (Ba, Ce, and Zr) were uniformly distributed, consistent with previous reports (Figure S3a,b) [46]. On the surface of BZCYYb1711 electrolyte after removal of PBSCF‐1711 skeleton, four points were selected for analysis: a clean surface (point 1), a Y and Yb enrichment region (point 2), and nanoparticles at grain boundaries (points 3 and 4) (Figure 4d). The results indicate that the element distribution on the clean surface (point 1) closely matches the stoichiometric ratio of BZCYYb1711. Segregation of Y and Yb elements was observed at point 2, consistent with findings in Figure S3a,b. Additionally, Pr, Sr, Co and Fe elements were detected at points 3 and 4, indicating the successful deposition of PBSCF nanoparticles into BZCYYb1711 skeleton and electrolyte surface by the nitrate solution impregnation. The EDX scanning source data for points 1–4 in Figure 4d are provided in Figure S3d.

**FIGURE 5:**
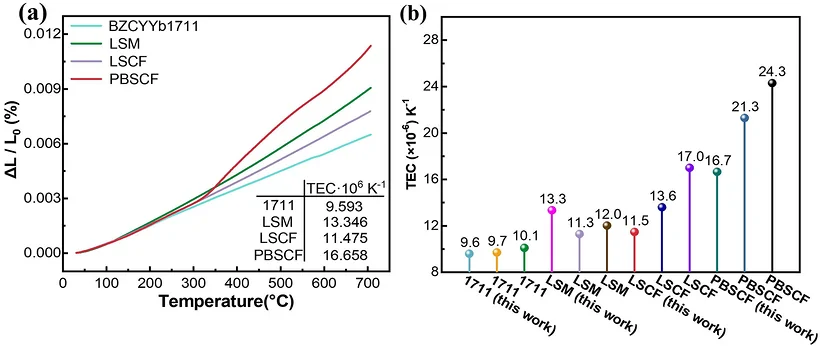
(a) Linear thermal expansion of BZCYYb1711, LSM, LSCF, and PBSCF during the heating process within the temperature range of 30–700°C in air. (b) Comparison of thermal expansion coefficients (TECs) of BZCYYb1711, LSM, LSCF, and PBSCF with values reported in the literature.

Figure 5a shows the plots of linear thermal expansion curves of BZCYYb1711, LSM, LSCF, and PBSCF. For PBSCF, ΔL/L_0_ is not strictly linearly dependent on temperature, with an obvious inflection point observed at 325°C. Jiang et al. reported an inflection point at 250°C for PBSCF previously [15]. The TEC values for BZCYYb1711, LSM, LSCF, and PBSCF were 9.59 × 10^−6^ K^−1^, 13.35 × 10^−6^ K^−1^, 11.48 × 10^−6^ K^−1^ and 16.66 × 10^−6^ K^−1^ in air, respectively. A comparison between TECs test results and literature‐reported values is listed in Figure 5b and Table S2 [15, 29, 31, 47, 48, 49, 50]. The TEC of BZCYYb1711 prepared by gel‐casting method is 9.59 × 10^−6^ K^−1^, smaller than that reported in the literature and the TECs of the powder obtained by direct calcination of the nitrate solution are also smaller (except for LSM) (Figure 5b). Similar and matching TECs can reduce the occurrence of delamination and cracks during the operation of PCFCs. In this study, the same material as the electrolyte, BZCYYb1711, was selected as the cathode skeleton, and cathodic active components such as LSM, LSCF, and PBSCF were introduced into the skeleton via nitrate impregnation. Due to the low impregnation density, the TEC of the impregnated nanocomposite cathode is similar to the electrolyte. Li et al. also anchored Co particles on the surface of the BZCY skeleton through reduction exsolution. The TEC of the R‐BCZY‐Co_x_ composite electrodes (x = 0.1, 0.2, 0.3) range from 10.2 × 10^−6^ K^−1^ to 11.4 × 10^−6^ K^−1^, close to 9.6 × 10^−6^ K^−1^ of pure BCZY electrolyte [23].

### 2.3 Electrochemical Performance

**FIGURE 6:**
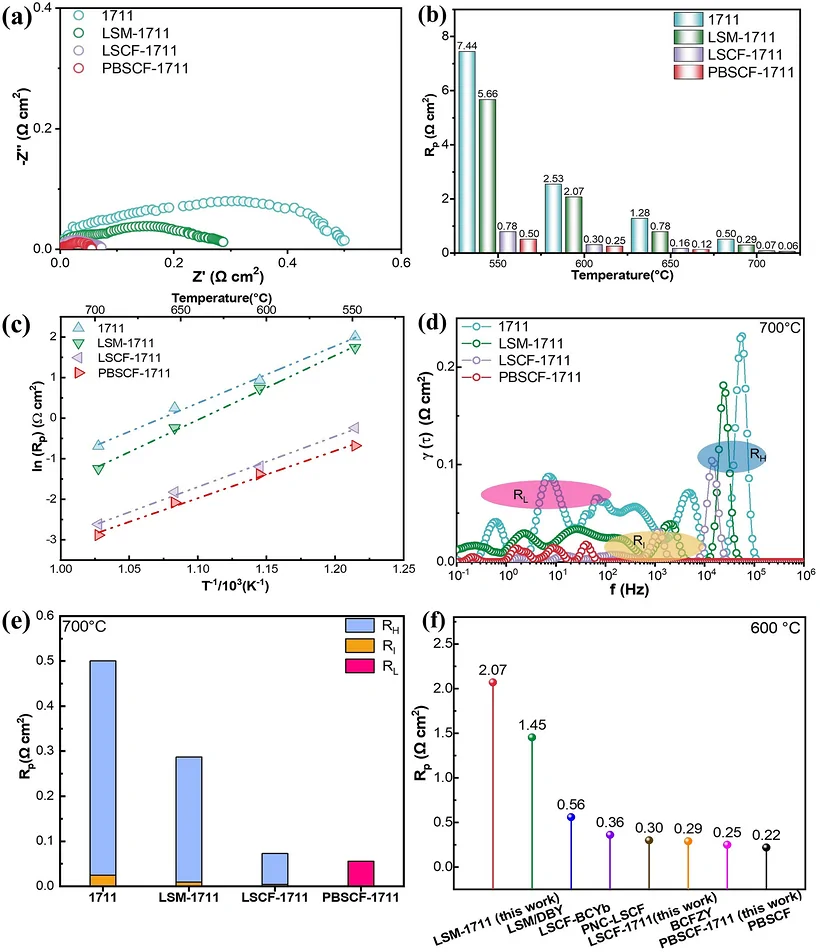
(a) EIS results of symmetric cells with 1711, LSM‐1711, LSCF‐1711, and PBSCF‐1711 cathodes measured in wet air at 700°C and the R_o_ was subtracted for clear comparison; (b) Comparison of R_p_ measured in wet air at 550–700°C; (c) Activation energy plots; (d) DRT curves measured in wet air at 700°C; (e) Comparison of R_p_ values (including R_H_, R_I_, and R_L_); and (f) Comparison of R_p_ of LSM‐1711, LSCF‐1711, and PBSCF‐1711 with other representative LSM/DBY [51], LSCF‐BCYb [52], PNC‐LSCF [28], BCFZY [53], and PBSCF [54] cathodes reported in the literature.

The electrochemical performance of impregnated nanocomposite electrodes as well as pristine BZCYYb1711 skeleton was examined using symmetrical cells and the results are given in Figure 6. The superior cathode materials for PCFCs demonstrate high electronic conductivity, excellent ORR catalytic activity, ionic conductivity for both O^2−^ and H^+^, and substantial porosity to facilitate the transport of oxidizing gases. For pristine BZCYYb1711 skeleton (1711 as shown in Figure 6a), the impedance responses are characterized by a large and depressed arc with the electrode polarization resistance (R_p_) of 0.50 Ω cm^2^ at 700°C and ohmic resistance (R_o_) of 4.67 Ω cm^2^ (Figure S5a). The large R_p_ and R_o_ values indicate that pristine BZCYYb1711 skeleton (1711) is not active for the ORR. This is understandable as pure BZCYYb1711 is mainly proton conductive with no electronic conductivity. Following impregnation with nitrate, the deposition of active nanoparticles on the plate‐like particles within the skeleton and on the electrolyte surface occurs (Figure 4a), enhancing the electronic conductivity as well as the active sites for ORR. As a result, the impregnated cathode exhibits lower R_p_ and R_o_. For instance, the R_p_ and R_o_ values of LSM‐1711 electrode were 0.29 and 2.43 Ω cm^2^, respectively (Figure 6b and Figure S5b). The much lower electrode ohmic resistance, R_o_ is clearly due to the fact that LSM is a well‐known excellent electronic conductor. However, the performance of the impregnated 1711 nanocomposite electrodes is strongly dependent on the impregnated active components. For example, for BZCYYb1711 skeleton impregnated with LSCF and PBSCF, the R_p_ values of LSCF‐1711 and PBSCF‐1711 are 0.78 and 0.50 Ω cm^2^ at 550°C, substantially lower than 5.66 Ω cm^2^ obtained on LSM‐1711 under identical conditions (Figure 5b). This is due to the fact that LSCF is a mixed electronic and ionic conductor (MIEC), while PBSCF is a triple conducting oxide (TCO). The PBSCF's triple‐conducting capability (H^+^/O^2−^/e^−^) enables the oxygen reduction reaction to occur across its entire bulk and surface, rather than being confined to the three‐phase boundary (TPB) areas. This demonstrates that impregnation of active components effectively enhances the electrochemical performance of BZCYYb1711 skeleton. The activation energy of LSCF‐1711 and PBSCF‐1711 electrodes is 1.07 and 1.01 eV, also significantly lower than 1.37 and 1.21 eV measured on LSM‐1711 electrode and pristine 1711 skeleton, respectively. This shows that increasing the number of reactive sites can effectively reduce the activation energy of the cathodes for ORR.

To further investigate the effect of impregnation, the distribution of relaxation times (DRT) was employed to analyze R_p_. According to the DRT analysis reported by Chen et al., the DRT curve can be subdivided into three electrochemical processes. The high‐frequency region (R_H_) corresponds to the charge transfer process, while the intermediate‐frequency (R_I_) and low‐frequency (R_L_) regions correspond to oxygen surface exchange and gas diffusion, respectively [55]. Impregnation effectively accelerates charge transfer and oxygen surface exchange, resulting in a significant decrease in R_H_ and R_I_ (Figure 6d). This phenomenon can be attributed to the deposition and formation of electronic, oxide ion and/or proton conducting nanoparticles within the skeleton structure, which facilitates ionic species transportation as well as surface diffusion and exchange processes (Figure 4). Furthermore, the oxide and perovskite nanoparticles generated through impregnation create more reactive sites for ORR. As shown in Figure 6e the reaction on 1711, LSM‐1711 and LSCF‐1711 electrodes is primarily dominated by the steps associated with high frequency region, R_H_, corresponding to the charge transfer process. In contrast, the reaction rate of ORR on PBSCF‐1711 cathode predominantly stems from the low frequency region, R_L_ corresponding to the gas diffusion process. This evidently indicates that impregnation of TCO components such as PBSCF can effectively reduce change transfer and surface exchange resistances of ORR. This is also supported by the excellent R_p_ value of 0.25 Ω cm^2^ at 600°C in wet air measured on PBSCF‐1711 electrode in this study, which is lower than those of reported several representative cathodes, including 1.45 Ω cm^2^ on La_0.8_Sr_0.2_MnO_3‐δ_‐Dy_0.16_Y_0.08_Bi_1.76_O_3‐δ_ (LSM‐DBY) [51], 0.56 Ω cm^2^ on La_0.6_Sr_0.4_Fe_0.8_Co_0.2_O_3‐δ_‐BaCe_0.9_Yb_0.1_O_3‐δ_ (LSCF‐BCYb) [52], 0.36 Ω cm^2^ on La_0.6_Sr_0.4_Fe_0.8_Co_0.2_O_3‐δ_‐Pr_2_Ni_0.5_Co_0.5_O_4‐δ_ (PNC‐LSCF) [28] and 0.29 Ω cm^2^ on BaCo_0.4_Fe_0.4_Zr_0.1_Y_0.1_O_3‐δ_ (BCFZY) [53], is also comparable with 0.22 Ω cm^2^ reported on pure PBSCF [54] (Figure 6f).

To directly verify the involvement of proton‐related kinetics and substantiate the peak assignments, Figure S10 shows the EIS results and DRT analysis of LSCF‐1711 cathode under varying water vapor partial pressures conditions measured at 500°C. The impedance corresponding to the high‐frequency range (R_H_) does not change significantly. However, the impedance corresponding to the intermediate‐frequency (R_I_)) and especially the low‐frequency (R_L_) decreases sharply with an increase in water vapor pressure, which is more likely associated with the process of the hydrated proton formation or/and transfer [56].

**FIGURE 7:**
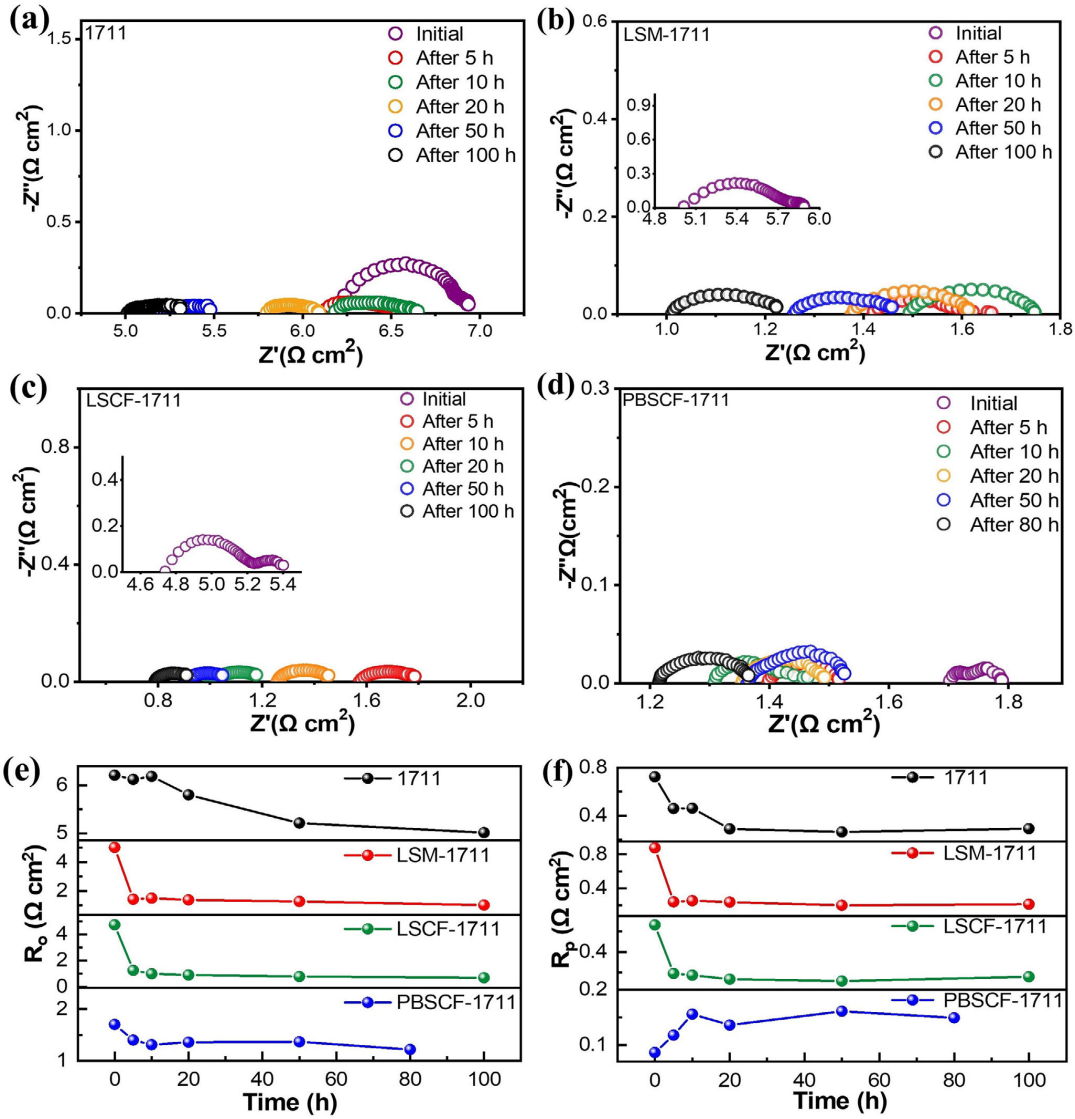
EIS results of 1711(a), LSM‐1711(b), LSCF‐1711(c) and PBSCF‐1711(d) cathodes, measured at 700°C in wet air as a function of polarization time under a current density of 0.5 A cm^−2^, and the corresponding plots of R_o_ (e) and R_p_ (f) with the polarization time. The tests were done in a three‐electrode‐cell arrangement.

The stability of impregnated nanocomposite electrodes was evaluated at 700°C in wet air under a current density of 0.5 A cm^−2^, using constant‐current polarization and electrochemical impedance spectroscopy (EIS), and the results are shown in Figure 7. For the unimpregnated 1711 cathode (Figure S6a), the initial polarization voltage reached 2.3 V and slightly decreased to 2.1 V after 100 h, indicating persistently high reaction resistance. For impregnated cathodes, the initial overpotential was substantially lower, and stability was significantly improved. Using LSM‐1711 as an example, the initial voltage decreased sharply from 1 V to 0.7 V within 1 h, and the voltage was stabilized at 0.52 V after 100 h (Figure S6b). The voltage ranges of LSCF‐1711 and PBSCF‐1711 were 0.74–0.4 V and 0.68–0.64 V, respectively (Figure S6c,d). The pristine 1711 and LSM‐1711 cathode exhibited larger R_p_ (0.724 and 0.876 Ω cm^2^, respectively) and R_o_ (6.212 and 5.583 Ω cm^2^, respectively) (Figure 7a,b). In comparison, the initial R_p_ and R_o_ values of the PBSCF‐1711 cathode were much lower, 0.086 and 1.70 Ω cm^2^, respectively (Figure 7c,d). It is noteworthy that the R_p_ and R_o_ values of all cathodes decreased significantly after 2–5 h of polarization. For instance, the LSCF‐1711 cathode demonstrated a decrease in R_p_ from 0.664 to 0.206 Ω cm^2^, while the R_o_ value decreased from 4.740 to 1.579 Ω cm^2^ within 2 h polarization (Figure 7e,f). The R_p_ and R_o_ values of all cathodes stabilized after 10 h of polarization, except for the R_o_ value of the 1711 cathode, which continued to decline until reaching 5.016 Ω cm^2^. Notably, the R_p_ of PBSCF‐1711 exhibited minimal variation during the long‐term time test, consistent with its stable polarization voltage (Figure S6d). Tao et al. also reported the infiltration of a Co‐Fe catalyst into ammonia‐fed protonic ceramic fuel cells. After 50–80 h of polarization under current densities of 268, 446 and 625 mA cm^−2^, the R_o_ value of the cells remained around 0.24 Ω cm^2^, while the R_p_ value decreased significantly from 0.71 to 0.32 Ω cm^2^. The improvement in electrochemical performance was attributed to enhanced proton conduction and molecular adsorption/dissociation on the anode surface [57].

**FIGURE 8:**
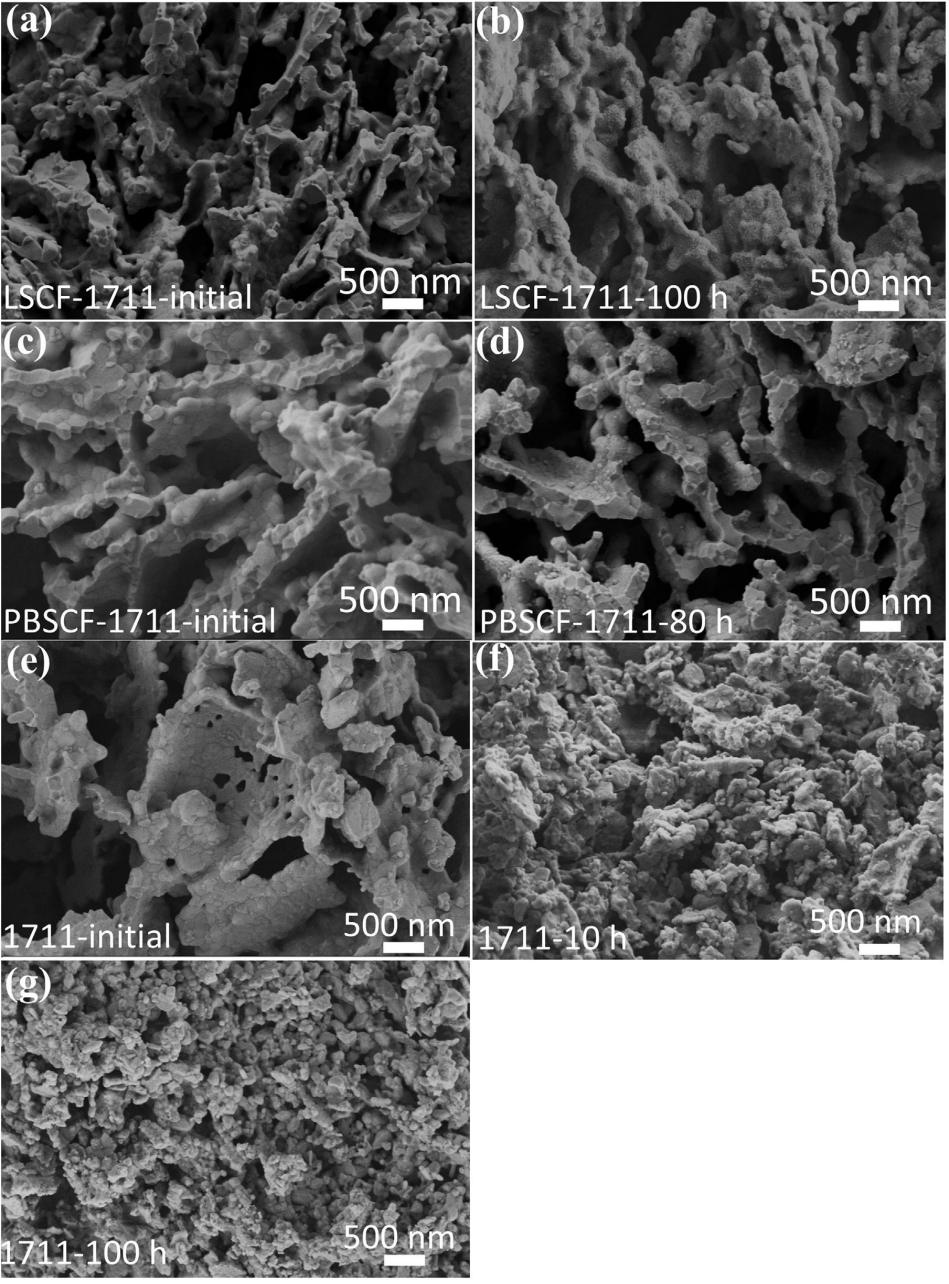
Cross‐section SEM images of LSCF‐1711(a,b), PBSCF‐1711(c,d) and 1711 (e–g) skeleton before and after constant current polarization under density of 0.5 A cm^−2^ in a three‐electrode‐cell arrangement.

To further investigate the reasons for the decrease in both R_o_ and R_p_, the microstructure of 1711, LSCF‐1711 and PBSCF‐1711 skeletons before and after constant current polarization was examined and the results are shown in Figure 8. For LSCF‐1711, the initial microstructure is characterized by an interconnected plate‐like network framework with uniformly distributed pores (Figure 8a), similar to that of the pristine 1711 skeleton structure formed at 1100°C (Figure 1f). After 100 h polarization, the porous morphology of the skeleton remains more or less the same, demonstrating satisfactory structural stability (Figure 8b). For PBSCF‐1711, the initial highly porous skeleton morphology remains almost unchanged after 80 h of polarization (Figure 8c,d), due to the characteristics of TCOs (conducting H^+^, e^−^ and O^2−^), which effectively reduce the overpotential (0.68 V) (Figure S6d). The voltage variation and impedance change of PBSCF‐1711 were minimal during the polarization process. It should be noted that nano‐agglomerated particles appeared on the surface of particles under the influence of polarization in wet air on both LSCF‐1711 and PBSCF‐1711 skeletons (Figure 8b,d). On the other hand, the pristine 1711 skeleton structure changes significantly after polarization. After polarization at 0.5 A cm^−2^ for only 10 h, the fully interconnected and plate‐like three‐dimensional network skeleton structure collapsed and disintegrated into fragmented round‐shaped as well as small plate‐like particles with substantially reduced interconnectivity (Figure 8e,f). With further increase of polarization time to 100 h, the original interconnected plate‐like network structure completely disappears and fractures into round‐shaped particles and debris (Figure 8g). Most interestingly, the BZCYYb1711 skeleton after polarization treatment at 0.5 A cm^−2^ for 100 h is microstructurally very similar to that after sintering at 1200°C (Figure 1f). Thus, such pronounced microstructure transformation can be attributed to the exclusive proton conduction mechanism of BZCYYb1711, which lacks electronic conductivity. The high initial overpotential (2.5 V) (Figure S6a), required to drive electron transfer across the insulating framework, generates substantial Joule heating. This thermal effect induces rather fast structural degradation during the polarization period. For both LSCF‐1711 and PBSCF‐1711, their ability to conduct electrons, oxygen ions and/or protons significantly lowers the overpotential, minimizing Joule heating and preserving structural stability.

The current study evidently shows that the interconnected plate‐like network is thermodynamically an unstable structure and can be deteriorated into fragmented, round‐shaped and agglomerated particles with no or substantially reduced interconnectivity. Impregnation of electrochemically active species such as LSCF and PBSCF with high electron, oxygen ion and/or proton conductivities can enhance the charge transfer process, suppress overpotential and thus reduce substantially the associated Joule heating and minimizing the detrimental effect of polarization on the skeleton microstructure and interconnectivity.

### 2.4 Stability and Cyclic Electrochemical Performance

**FIGURE 9:**
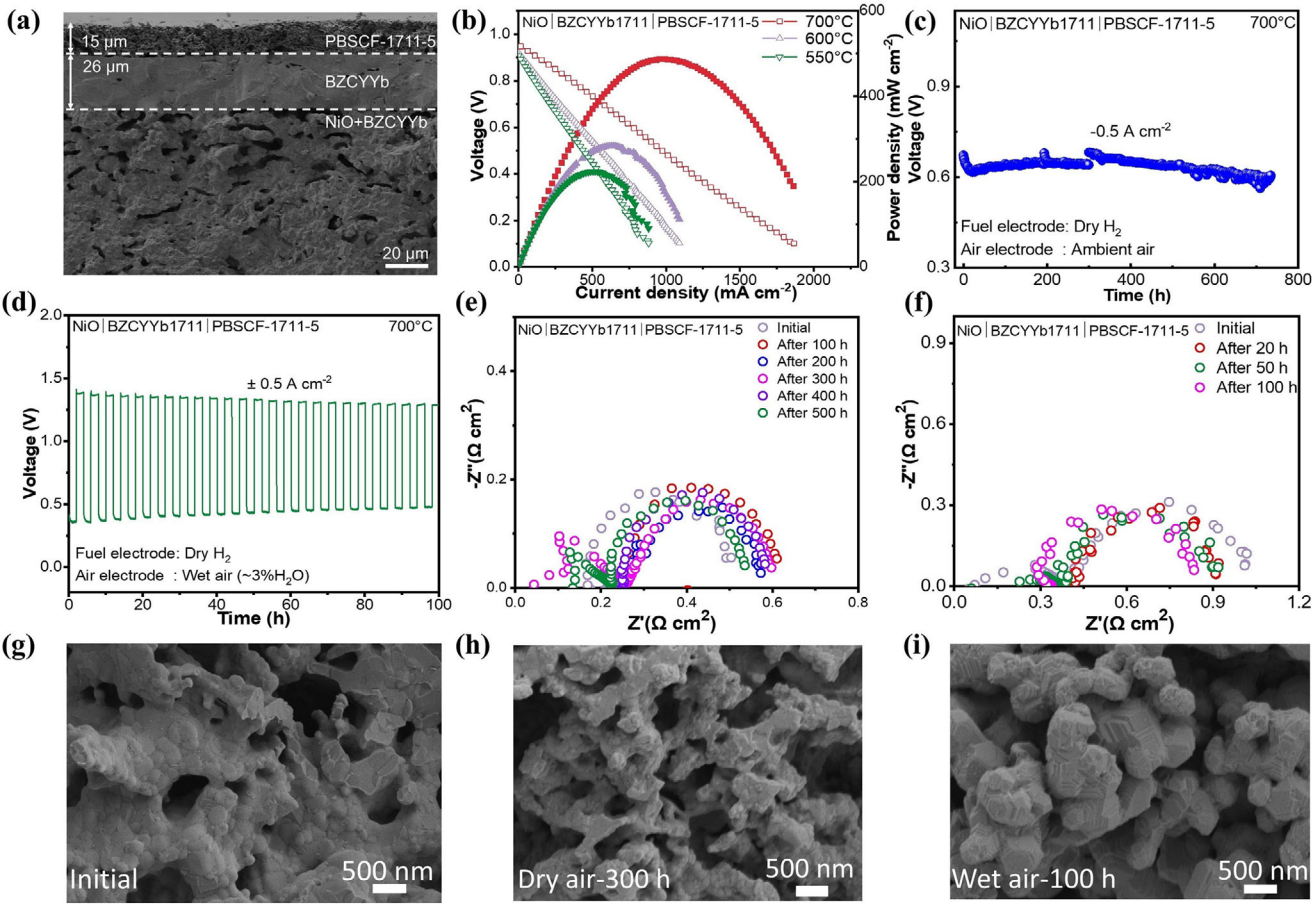
(a) Cross‐sectional SEM image of single cell with Ni+BZCYYb anode supports, thin BZCYYb1711 electrolyte and PBSCF‐1711‐5 (with loading of 25 mg cm^−2^) air electrode, (b) Current‐voltage‐power (I‐V‐P) curves of the single cell, (c) Stability of the single cell tested at 700°C for 734 h under a constant current density of 0.5 A cm^−2^ in H_2_/air, (d) Reversible operation of the cell at a current density of 0.5 A cm^−2^ at 700°C, switching at an interval of 2 h between the fuel cell (FC) and electrolysis cell (EC) operation modes. (e) EIS results of the single cell during 734 h in FC mode. (f) EIS results of the single cell during reversible cycling operation. Cross‐section SEM images of (g) PBSCF‐1711‐5 before test, (h) after 300 h of operation in FC mode and (i) after 100 h of reversible cycling operation.

The electrochemical performance stability and reversible operation capability of the air electrode formed by the nitrate impregnated BZCYYb1711 skeleton were further evaluated using fuel‐electrode‐supported cells with PBSCF‐1711‐5 air electrode. The single cell consisted of a Ni‐BZCYYb‐fuel‐electrode‐supporter, a dense BZCYYb1711 electrolyte (∼26 µm), and a porous PBSCF‐1711 air electrode (∼15 µm) (Figure 9a). The cell with PBSCF‐1711 air electrode achieved a peak power density (PPD) of 486 mW cm^−2^ at 700°C, while the cell with unimpregnated BZCYYb1711 skeleton electrode achieved only 13 mW cm^−2^ (Figure 9b and Figure S7a). The cell also showed excellent long‐term durability under FC operation mode under the current density of −0.5 A cm^−2^ at 700°C for 734 h (Figure 9c). The initial R_p_ of the cell was 0.33 Ω cm^2^ and increased slightly to 0.39 Ω cm^2^ after polarization at 700°C and 0.5 A cm^−2^ in dry H_2_/air for 500 h (Figure 9e). The cell voltage decreased from an initial value of 0.672 to 0.606 V after polarization for 734 h, exhibiting a voltage degradation rate of 0.013% h^−1^, which is lower than those of reported several representative air electrodes in PCFCs (Table S3) [28, 58, 59, 60, 61, 62]. The strategy of electrolyte‐electrode material homology effectively addressed the problem of TECs mismatch, significantly reducing the voltage decay rate (Figure 5).

Additionally, a cycling test was performed on the cell with PBSCF‐1711 air electrode at 700°C under a constant current density of 0.5 A cm^−2^, alternating between fuel cell (FC) and electrolysis cell (EC) modes at 2 h intervals with dry H_2_ in the fuel side and wet air in the air side of the cell (Figure 9d). At the initial stage of the polarization, the cell with PBSCF‐1711‐5 air electrode showed a cell voltage of 0.37 V under FC operation mode and 1.38 V under EC operation mode, exhibiting a cell voltage difference of 1.01 V. After polarization for 100 h with 25 cycles, the cell voltage stabilized at 0.48 V under FC operation mode and 1.29 V under EC operation mode, corresponding to a reduced voltage difference of 0.81 V. Meanwhile, the R_p_ value of the cell with PBSCF‐1711‐5 air electrode decreased from 0.94 to 0.51 Ω cm^2^ at the end of the polarization test (Figure 9f). The voltage change should be interpreted together with the corresponding impedance evolution, rather than as direct evidence of improved performance. Marrony et al. also reported that the voltage in FC mode gradually decreased, whereas that in EC mode gradually increased during cycling, eventually approaching an asymptotic state [63]. In addition, the peak power density (PPD) of 486 mW cm^−2^ at 700°C is moderate compared with the reported PCFCs recently, but it remains meaningful in view of the long‐term stability and reversible FC/EC operation demonstrated here. The relatively thick BZCYYb1711 electrolyte and the low impregnation density are likely to constrain the peak power density.

The microstructure of the PBSCF‑1711‑5 air electrodes after 300 h of operation in FC mode in dry air and after 100 h of cyclic testing in FC/EC in wet air was examined. The pristine PBSCF‑1711‑5 electrode shows a typical well‑interconnected skeleton network with uniform deposition of particles on the surface of plate‐like particles (Figure 9g). After polarization at 0.5 A cm^−2^ in dry air for 300 h, the plate‐like and interconnected skeleton framework remained intact with the reduced size of plate‐like particles and the increase in porosities (Figure 9h). The smaller plate‐like particles and increased porosity within the skeleton structure enhances the gas transport and also the active sites and contact with the electrolytes, indicated by the decrease in cell ohmic resistance (R_o_) from 0.17 to 0.05 Ω cm^2^ (Figure 9e). After 100 h of cyclic polarization in FC/EC operation in wet air, there is a clear microstructural change from plate‐like network structure to round‐shaped particle network structure (Figure 9i). In addition, Figure S9 presents cross‐sectional SEM images of the single cell with PBSCF‐1711‐5 as the air electrode before and after long‐term polarization tests. After 300 h of operation in dry air and 100 h in wet air, the interface between air electrode and electrolyte remains well bonded, demonstrating good interfacial stability.

Overall, during the polarization process in FC operation, the electrode microstructure evolves from a plate‐like network into a more open three‐dimensional porous framework to enlarge gas‐transport channels, thereby facilitating oxygen supply and water removal, leading to a reduction in R_o_ (Figure 9e). During 100 h of cyclic polarization in FC/EC operation, the plate‐like structure further transforms into interconnected round‐shaped particles. While maintaining structural integrity, this evolution promotes gas diffusion and exposes more active sites for both ORR in fuel‑cell mode and the oxygen evolution reaction (OER) in EC mode, leading to a reduction in R_p_ (Figure 9f). These microstructural changes result in improved electrochemical performance of the impregnated composite PCFC cathode during polarization.

## 3 Conclusions

In this work, we systematically investigate the formation of stable and interconnected BZCYYb1711 skeleton on BZCYYb1711 electrolyte surface at different sintering temperatures (900‐1200°C) and summarize the results of the skeleton formation, interconnectivity and interface. Based on this work, the best condition for the formation of interconnected and stable BZCYYb1711 skeleton structure on the BZCYYb1711 electrolyte with excellent interface is the sintering at 1100°C for 2 h in air. Sintering at temperature lower than 1100°C leads to an insufficiently interconnected BZCYYb1711 skeleton structure, while sintering at temperature higher than 1100°C results in the collapses and disintegration of the three‐dimensional plate‐like skeleton structure and significant coarsening of the interface between the skeleton and the electrolyte. There is a close relationship between the interconnectivity and skeleton structure formation and sintering temperature, most likely governed by thermodynamics of densification of Ba‐containing electrolyte ceramics.

Based on the BZCYYb1711 skeleton formed, we selected three representative cathode materials (LSM, LSCF and PBSCF) then introduced them into the porous BZCYYb1711 skeleton by nitrate impregnation, forming nanocomposite electrodes for PCFCs. The results indicate that the impregnated components formed stable perovskite phases and were uniformly distributed as nanoparticles on BZCYYb1711 skeleton surface. Electrochemical tests revealed that the impregnation effectively reduced both the R_p_ value and activation energy. Among the three cathodes, PBSCF‐1711 cathode exhibited the best performance, with a R_p_ value of 0.06 Ω cm^2^ at 700°C in wet air and activation energy of 1.01 eV, significantly lower than 1.37 eV, obtained on LSM‐1711 nanocomposite cathode due to the TCO properties of the impregnated PBSCF phase.

The Long‐term stability tests under a three‐electrode system confirmed that cathodes after impregnation exhibited excellent steam tolerance over 100 h of operation. More importantly, excellent operational stability was demonstrated, with stable operation sustained for 734 h in FC mode and 100 h under cycling testing in FC/EC operation conditions. Although the peak power density of the single cell (486 mW cm^−2^ at 700°C) is moderate compared with the highest reported PCFC values, this study demonstrates that preparing a skeleton with the same material as the electrolyte and introducing the electrochemical active materials via nitrate impregnation is a simple, reproducible, and effective strategy for developing nanocomposite cathodes for intermediate‐temperature protonic ceramic cells. This can effectively address mismatch of TECs, thereby enhancing mechanical and long‐term electrochemical stability (with a low voltage decay rate of 0.013% h^−1^). The results also demonstrate that the electrochemical activity and stability of the impregnated nanocomposite are closely related to that nature of the impregnated active components.

## 4 Experimental Section

### 4.1 BZCYYb1711 Skeleton and Cell Fabrication

The electrolyte powder BaZr_0.1_Ce_0.7_Y_0.1_Yb_0.1_O_3‐δ_ (BZCYYb1711) was synthesized by a gel‐casting technique, as described in our previous work [46]. To prepare electrolyte‐supported symmetric cells, 0.5 g of BZCYYb1711 powder was uniformly distributed in a 15 mm diameter mold, subsequently pressed at 200 MPa for 1 min, and calcined at 1600°C for 5 h to obtain a dense electrolyte disc. For the skeleton fabrication, BZCYYb1711 powder was mixed with a Polyvinyl Butyral binder (PVB) at a 6:4 ratio to form a slurry. The slurry was screen‐printed onto both sides of electrolyte disc, followed by sintering at 900, 1000, 1100, and 1200°C for 2 h to investigate the optimal formation temperature of BZCYYb1711 skeleton.

Single cells with NiO‐BZCYYb1711 as the anode‐support and thin BZCYYb1711 electrolyte structure were fabricated through dry pressing and high‐temperature co‐sintering. The anode‐support composition was NiO: BZCYYb1711: starch = 7:3:1, with polyvinyl butyral (PVB) added as a binder. The mixed powder was ball‐milled in ethanol for 24 h and subsequently dried at 80°C for 10 h. Approximately 0.35 g of anode powder was pressed into a pellet with a diameter of 15 mm under 100 MPa pressure, followed by uniform deposition of 0.03 g BZCYYb1711 electrolyte on the anode surface and co‐pressing at the same pressure. The bilayer BZCYYb1711 electrolyte pellet was sintered in air at 1450°C for 5 h. The BZCYYb1711 skeleton was formed on the BZCYYB1711 electrolyte side with an effective area of 0.28 cm^2^, as shown above.

### 4.2 Impregnation

Pr(NO_3_)_3_·6H_2_O, Ba(NO_3_)_2_, Sr(NO_3_)_2_·6H_2_O, Co(NO_3_)_2_·6H_2_O and Fe(NO_3_)_3_·9H_2_O (all from Aladdin) were dissolved in deionized water according to the stoichiometric ratio of PrBa_0.5_Sr_0.5_Co_1.5_Fe_0.5_O_5+_ *_δ_* to form a 0.1 M nitrate impregnation solution. Glycine was used as a chelating agent, with a molar ratio of glycine to nitrate of 1.5. A 5 µL aliquot of the impregnation solution was deposited onto the as‐prepared BZCYYb1711 skeleton using a microliter syringe. The sample was dried in an oven at 50°C for 1 h and subsequently sintered at 1100°C for 2 h to form stable phases, and the resulting nanocomposite electrode was named as PBSCF‐1711. Similarly, nitrate solutions of (La_0.8_Sr_0.2_)MnO_3‐δ_ (LSM) and La_0.6_Sr_0.4_Co_0.2_Fe_0.8_O_3‐δ_ (LSCF) were prepared and impregnated onto as‐prepared BZCYYb1711 skeleton. After sintering at 1100°C for 2 h, the formed nanocomposite electrodes were named as LSM‐1711 and LSCF‐1711, respectively. The loading of the impregnated PBSCF, LSM and LSCF phase in the skeleton was estimated to be 5 mg cm^−2^, based on the weight difference before and after impregnation treatment. An additional PBSCF‐1711 electrode with a high loading of 25 mg cm^−2^ was prepared by repeating the impregnation steps for single cell tests which was named as PBSCF‐1711‐5. To determine the phase composition, the nitrate impregnation solution was sintered at 1100°C for 2 h and the resulting powder was subjected to XRD analysis.

### 4.3 Electrochemical Testing

For symmetric cell, silver paste was uniformly applied to both surfaces as a current collection layer and connected with a silver wire. For three‐electrode cell, silver paste was coated on the side of the nanocomposite cathode to serve as the working electrode (WE). A silver wire was placed around the opposite side of the cell and secured as the reference electrode (RE), while another silver wire was fixed at the center of the same side as the counter electrode (CE). The atmosphere used for symmetric cell and three‐electrode cell tests was wet air by passing the air through a water bottle at room temperature (∼3% H_2_O/air) and the flow rate was 20 mL min^−1^. For fuel‐cell tests, dry hydrogen was introduced into the fuel electrode side while the air electrode was exposed to ambient air. For the electrolysis‐cell tests, the fuel electrode was fed with dry hydrogen, and the air electrode was fed with wet air (∼3% H_2_O/air). The flow rate of both H_2_ and wet air for single cell tests was 60 mL min^−1^. All Electrochemical impedance spectroscopy (EIS) tests were conducted under open‐circuit conditions with an amplitude of 10 mV in a frequency range of 0.1–10^6^ Hz by using a Solartron 1260A electrochemical workstation. Testing was performed over a temperature range of 550–700°C.

### 4.4 Characterization

The interface between BZCYYb1711 skeleton and BZCYYb1711 electrolytes sintered at different temperatures was investigated by a field emission scanning electron microscopy (FE‐SEM, Sirion 200) and the corresponding elemental distribution was characterized by energy‐dispersive X‐ray spectroscopy (EDX, JXA‐iHP200F). Phase of nanocomposite electrodes and powder from the impregnation solution was measured using an X‐ray diffractometer (XRD, Rigaku MiniFlex600) with Cu‐K_𝛼_ radiation, scanning from 20 to 80° at a scan rate of 5° min^−1^. The microstructure and morphology of the skeleton, electrodes and cells were examined using a FE‐SEM and the corresponding elemental distribution was characterized by EDX. The respective diameter sizes were measured by ImageJ software. To determine the TECs of the skeleton and the impregnation component, BZCYYb1711 powder and the powders obtained by sintering the nitrate impregnation solution at 1100°C for 2 h were pressed into rectangular bars (20 mm in length, 6 mm in width and 4 mm in height). The precursor bars were then sintered at 1600°C (BZCYYb1711) and 1100°C (LSM, LSCF and PBSCF) respectively to obtain dense rectangular bars for testing by using a NETZSCH DIL 402CL dilatometer from 30 to 700°C in air at a heating rate of 10°C min^−1^. The TECs were calculated from the thermal variation of lattice parameters, i.e., the slope of (L‐L_0_)/L_0_ vs. Temperature. The slurry for the screen‐printed BZCYYb1711 skeleton was sintered at 1100°C for BET characterization using N_2_ adsorption‐desorption measurements on a Rize‐1020 surface area and porosity analyzer.

## Funding

This work was supported by the Guangdong Basic and Applied Basic Research Foundation (2023A1515111015), Foshan Xianhu Laboratory Young Fellowship Program (XHQN2022‐010), Foshan Xianhu Laboratory Innovation Program (XHRD2023‐001), Jilin Province science and technology development plan project (20240101092JC) and Innovative Research Team Project (CXTD2023009) introduced by Zhongshan Institute of Changchun University of Science and Technology.

## Conflicts of Interest

The authors declare no conflicts of interest.

## Supporting information




**Supporting File 1**: smll74677‐sup‐0001‐SuppMat.docx.

**Supporting File 2**: smll74677‐sup‐0002‐FigureS1‐S11.zip.

## Data Availability

The data that support the findings of this study are available from the corresponding author upon reasonable request.

## References

[1] A. B. Stambouli and E. Traversa , “Solid Oxide Fuel Cells (SOFCs): A Review of an Environmentally Clean and Efficient Source of Energy,” Renewable and Sustainable Energy Reviews 6 (2002): 433–455.

[2] S. P. Jiang , “Solid‐State Electrochemistry and Solid Oxide Fuel Cells: Status and Future Prospects,” Electrochemical Energy Reviews 5 (2022): 21.

[3] J. Cao , Y. Ji , and Z. Shao , “Perovskites for Protonic Ceramic Fuel Cells: A Review,” Energy & Environmental Science 15 (2022): 2200–2232.

[4] N. Tsvetkov , D. Kim , I. Jeong , et al., “Advances in Materials and Interface Understanding in Protonic Ceramic Fuel Cells,” Advanced Materials Technologies 8 (2023): 2201075.

[5] E. D. Wachsman and K. T. Lee , “Lowering the Temperature of Solid Oxide Fuel Cells,” Science 334 (2011): 935–939. 22096189 10.1126/science.1204090

[6] A. Dubois , S. Ricote , and R. J. Braun , “Benchmarking the Expected Stack Manufacturing Cost of Next Generation, Intermediate‐Temperature Protonic Ceramic Fuel Cells with Solid Oxide Fuel Cell Technology,” Journal of Power Sources 369 (2017): 65–77.

[7] C. Duan , R. Kee , H. Zhu , et al., “Highly Efficient Reversible Protonic Ceramic Electrochemical Cells for Power Generation and Fuel Production,” Nature Energy 4 (2019): 230–240.

[8] W. Zhang , X. Zhang , Y. Song , and G. Wang , “Recent Progress on Cathode Materials for Protonic Ceramic Fuel Cells,” Next Sustainability 3 (2024): 100028.

[9] G. C. Mather , D. Muñoz‐Gil , J. Zamudio‐García , J. Porras‐Vazquez , D. Marrero‐López , and D. Pérez‐Coll , “Perspectives on Cathodes for Protonic Ceramic Fuel Cells,” Applied Sciences 11 (2021): 5363.

[10] S. Dwivedi , “Solid Oxide Fuel Cell: Materials for Anode, Cathode and Electrolyte,” International Journal of Hydrogen Energy 45 (2020): 23988–24013.

[11] H. Uchida , S. Tanaka , and H. Iwahara , “Polarization at Pt Electrodes of a Fuel Cell with a High Temperature‐type Proton Conductive Solid Electrolyte,” Journal of Applied Electrochemistry 15 (1985): 93–97.

[12] B. K. Park , R. H. Song , S. B. Lee , et al., “A Perovskite‐Type Lanthanum Cobaltite Thin Film Synthesized via an Electrochemical Route and Its Application in SOFC Interconnects,” Journal of the Electrochemical Society 162 (2015): F1549–F1554.

[13] S. Guo , L. Jiang , Y. Li , et al., “From Electrolyte and Electrode Materials to Large‐Area Protonic Ceramic Fuel Cells: A Review,” Advanced Functional Materials 34 (2024): 2304729.

[14] H. Wang , X. Zhang , W. Zhang , et al., “Enhancing Catalysis Activity of La_0.6_Sr_0.4_Co_0.8_Fe_0.2_O_3‐_ * _δ_ * Cathode for Solid Oxide Fuel Cell by a Facile and Efficient Impregnation Process,” International Journal of Hydrogen Energy 44 (2019): 13757–13767.

[15] L. Jiang , T. Wei , R. Zeng , W.‐X. Zhang , and Y.‐H. Huang , “Thermal and Electrochemical Properties of PrBa_0.5_Sr_0.5_Co_2−x_Fe_x_O_5+δ_ (x = 0.5, 1.0, 1.5) Cathode Materials for Solid‐Oxide Fuel Cells,” Journal of Power Sources 232 (2013): 279–285.

[16] Z. Gao , X. Ding , D. Ding , L. Ding , S. Zhang , and G. Yuan , “Infiltrated Pr_2_NiO_4_ as Promising Bi‐Electrode for Symmetrical Solid Oxide Fuel Cells,” International Journal of Hydrogen Energy 43 (2018): 8953–8961.

[17] C. Xia , Y. Mi , B. Wang , B. Lin , G. Chen , and B. Zhu , “Shaping Triple‐Conducting Semiconductor BaCo_0.4_Fe_0.4_Zr_0.1_Y_0.1_O_3‐δ_ Into An Electrolyte for Low‐Temperature Solid Oxide Fuel Cells,” Nature Communications 10 (2019): 1707. 10.1038/s41467-019-09532-z PMC6461657 30979875

[18] Y. Kong , C. Sun , X. Wu , Y. Zhang , N. Zhang , and K. Sun , “CuCo_2_O_4_‐Er_0.4_Bi_1.6_O_3‐δ_: An Active Hybrid Cathode for Intermediate Temperature Solid Oxide Fuel Cells,” Ceramics International 45 (2019): 6037–6042.

[19] C. Liu , B. Ma , Z. Lin , Y. Zhou , and K. Wu , “Exploring(* _1‐x_ *) CuFe* _2_ *O* _4‐x_ *Gd* _0.1_ *Ce* _0.9_ *O* _1.95_ *Composites as the Cathode Materials for Solid Oxide Fuel Cells,” Materials Letters 325 (2022): 132860.

[20] K. Luo , M. Ma , Y. Yu , Y. Liu , D. Xiao , and Y. Guo , “In Situ Self‐Assembly of a High Active and Durable Composite Electrode for Protonic Ceramic Fuel Cells,” Ceramics International 50 (2024): 40586–40593.

[21] C. Peng , X. Han , S. Mabaleha , P. Kwong , Y. Zheng , and X. Xu , “Recent Advances in Perovskite Air Electrode Materials for Protonic Solid Oxide Electrochemical Cells,” Energy & Environmental Science 18 (2025): 4555–4595.

[22] T. Cao , O. Kwon , R. J. Gorte , and J. M. Vohs , “Metal Exsolution to Enhance the Catalytic Activity of Electrodes in Solid Oxide Fuel Cells,” Nanomaterials 10 (2020): 2445. 33297343 10.3390/nano10122445 PMC7762234

[23] Z. Li , M. Peng , Y. Zhao , J. Li , and Y. Sun , “Minimized Thermal Expansion Mismatch of Cobalt‐Based Perovskite Air Electrodes for Solid Oxide Cells,” Nanoscale 13 (2021): 20299–20308. 34846404 10.1039/d1nr06845h

[24] S. H. Woo , S.‐W. Baek , D. S. Park , et al., “Microstructural and Electrochemical Properties of Impregnated La_0.4_Sr_0.6_Ti_0.8_Mn_0.2_O_3±d_ Into a Partially Removed Ni SOFC Anode Substrate,” Journal of Alloys and Compounds 854 (2021): 157250.

[25] M. Wei , H. Chen , X. Zhao , et al., “High Performance La_2_NiO_4+_ * _δ_ * Impregnated La_0.6_Sr_0.4_Co_0.2_Fe_0.8_O_3‐_ * _δ_ * Cathodes for Solid Oxide Fuel Cells,” Solid State Ionics 387 (2022): 116065.

[26] X. Xi , X. Chen , G. Hou , N. Xu , Q. Zhang , and Z. Tao , “Fabrication and Evaluation of Sm_0.5_Sr_0.5_CoO_3−_ * _δ_ * Impregnated PrBaCo* _2_ *O* _5+δ_ * Composite Cathode for Proton Conducting SOFCs,” Ceramics International 40 (2014): 13753–13756.

[27] Y. Zhou , X. Meng , X. Ye , J. Li , S. Wang , and Z. Zhan , “Metal‐Supported Solid Oxide Fuel Cells with Impregnated SrFe_0.75_Mo_0.25_O_3_ Cathodes,” Journal of Power Sources 247 (2014): 556–561.

[28] P. Yao , J. Zhang , Q. Qiu , Y. Zhao , F. Yu , and Y. Li , “Enhancing Oxygen Reduction Kinetics and Proton Transfer of La,” Advanced Energy Materials 15 (2025): 2403335.

[29] Y. Sun , D. Jin , X. Zhang , et al., “Controllable Technology for Thermal Expansion Coefficient of Commercial Materials for Solid Oxide Electrolytic Cells,” Materials 17 (2024): 1216. 38473687 10.3390/ma17051216 PMC10933915

[30] Z. Li , B. Wei , Z. Lü , et al., “Evaluation of (Ba_0.5_Sr_0.5_)_0.85_Gd_0.15_Co_0.8_Fe_0.2_O_3−δ_ Cathode for Intermediate Temperature Solid Oxide Fuel Cell,” Ceramics International 38 (2012): 3039–3046.

[31] K. Nomura , H. Shimada , Y. Yamaguchi , W. Shin , Y. Okuyama , and Y. Mizutani , “Phase Transitions, Thermal Expansions, Chemical Expansions, and CO* _2_ * Resistances of Ba(Ce_0.8‐x_Zr_x_Y_0.1_Yb_0.1_)O_3‐_ * _δ_ * (x = 0.1, 0.4) Perovskite‐Type Proton Conductors,” Journal of The Electrochemical Society 169 (2022): 024516.

[32] N. Shah , T. Zhu , D. Feng , et al., “Fine‐tuning Thermal Expansion Characteristics of Solid Oxide Fuel Cell Cathode via Composite Cathode Fabrication,” Journal of Power Sources 616 (2024): 235143.

[33] H. Zheng , Y. Tian , L. Zhang , B. Chi , J. Pu , and L. Jian , “La_0.8_Sr_0.2_Co_0.8_Ni_0.2_O_3‐δ_ Impregnated Oxygen Electrode for H_2_O/CO_2_ Co‐Electrolysis in Solid Oxide Electrolysis Cells,” Journal of Power Sources 2018, 383, 93–101.

[34] J. Li , S. Wang , Z. Wang , R. Liu , T. Wen , and Z. Wen , “La_0.84_Sr_0.16_MnO_3−δ_ Cathodes Impregnated with Bi_1.4_Er_0.6_O_3_ for Intermediate‐Temperature Solid Oxide Fuel Cells,” Journal of Power Sources 2009, 194 (2), 625–630.

[35] S. P. Jiang , S. Zhang , Y. D. Zhen , and A. P. Koh , “Performance of GDC‐Impregnated Ni Anodes of SOFCs,” Electrochemical and Solid‐State Letters 7 (2004): A282.

[36] S. P. Jiang and S. H. Chan , “A Review of Anode Materials Development in Solid Oxide Fuel Cells,” Journal of Materials Science 39 (2004): 4405–4439.

[37] T. Okabe , Y. Nakazawa , Q. Lyu , T. Zhu , and N. Shikazono , “Ni‐Free Single GDC Anode Deposited on YSZ Porous Skeleton via Hydrothermal Treatment for High Humidity SOFC Operation,” Journal of Power Sources 653 (2025): 237713.

[38] M. Liu , J. Gao , X. Liu , and G. Meng , “High Performance of Anode Supported BaZr_0.1_Ce_0.7_Y_0.2_O_3−_ * _δ_ *(BZCY) Electrolyte Cell for IT‐SOFC,” International Journal of Hydrogen Energy 36 (2011): 13741–13745.

[39] J. Chen , W. Gao , L. Zhu , et al., “A Mixed Proton–Electron‐Conducting Cathode with a Ru Nanoparticle Catalyst for Electrochemical Ammonia Synthesis based on a Proton‐Conducting BZCYYb Electrolyte,” Journal of Materials Chemistry A 12 (2024): 26667–26677.

[40] G. Li , H. Jin , Y. Cui , L. Gui , B. He , and L. Zhao , “Application of a Novel (Pr_0.9_La_0.1_)_2_(Ni_0.74_Cu_0.21_Nb_0.05_)O_4+δ_‐Infiltrated BaZr_0.1_Ce_0.7_Y_0.2_O_3‐δ_ Cathode for High Performance Protonic Ceramic Fuel Cells,” Journal of Power Sources 341 (2017): 192–198.

[41] S. He , K. Chen , M. Saunders , et al., “Interface Formation and Mn Segregation of Directly Assembled La_0.8_Sr_0.2_MnO_3_ Cathode on Y_2_O_3_‐ZrO_2_ and Gd_2_O_3_‐CeO_2_ Electrolytes of Solid Oxide Fuel Cells,” Solid State Ionics 325 (2018): 176–188.

[42] S. He , M. Saunders , K. Chen , et al., “A FIB‐STEM Study of Strontium Segregation and Interface Formation of Directly Assembled La_0.6_Sr_0.4_Co_0.2_Fe_0.8_O_3‐_ * _δ_ * Cathode on Y_2_O_3_‐ZrO_2_ Electrolyte of Solid Oxide Fuel Cells,” Journal of The Electrochemical Society 165 (2018): F417–F429.

[43] T. Shimura , T. Hayashi , Y. Inaguma , and M. Itoh , “Magnetic and Electrical Properties of La_y_A_x_Mn_w_O_3_(A= Na, K, Rb, and Sr) With Perovskite‐Type Structure,” Journal of Solid State Chemistry 124 (1996): 250–263.

[44] J. Readman , A. Olafsen , Y. Larring , and R. Blom , “La_0.8_Sr_0.2_Co_0.2_Fe_0.8_O_3−δ_ as a Potential Oxygen Carrier in a Chemical Looping Type Reactor, An In‐Situ Powder X‐Ray Diffraction Study,” Journal of Materials Chemistry 16 (2005): 1931–1937.

[45] D. V. Karpinsky , I. O. Troyanchuk , K. Bärner , H. Szymczak , and M. Tovar , “Crystal Structure and Magnetic Ordering of the LaCo_1−x_Fe_x_O_3_ System,” Journal of Physics: Condensed Matter 17 (2005): 7219.

[46] J. Li , Z. Chen , Z. Yue , et al., “Effect of Synthesis Methods on the Sintering Behaviour of Proton‐Conducting Electrolyte Materials for Solid Oxide Fuel Cells,” Ceramics International 51 (2025): 50762–50778.

[47] I. M. Hung , D. Mohanty , Y.‐R. Lin , S.‐W. Lee , C.‐J. Tseng , and Y.‐W. Chen , “Synthesis and Electrochemical Performance of PrBa_0.5_Sr_0.5_Co_1.5_Fe_0.5_O_5+δ_ Double Perovskite Cathode Material for Solid Oxide Fuel Cell,” International Journal of Hydrogen Energy 145 (2025): 371–379.

[48] M. Mori , Y. Hiei , N. M. Sammes , and G. A. Tompsett , “Thermal‐Expansion Behaviors and Mechanisms for Ca‐ or Sr‐Doped Lanthanum Manganite Perovskites Under Oxidizing Atmospheres,” Journal of The Electrochemical Society 147 (2000): 1295.

[49] V. Vibhu , S. Yildiz , I. C. Vinke , R. A. Eichel , J. M. Bassat , and L. G. J. de Haart , “High Performance LSC Infiltrated LSCF Oxygen Electrode for High Temperature Steam Electrolysis Application,” Journal of The Electrochemical Society 166 (2019): F102–F108.

[50] Y. Xu , X. Guo , C. Lin , et al., “Thermal Properties and Microstructures Analysis of YSZ and YSZ‐Al* _2_ *O* _3_ * Thermal Barrier Coatings,” Journal of Thermal Spray Technology 29 (2020): 574–581.

[51] Q. Lin , L. Bian , C. Liu , et al., “Improved La_0.8_Sr_0.2_MnO_3‐_ * _δ_ * Oxygen Electrode Activity by Introducing High Oxygen Ion Conductor Oxide for Solid Oxide Steam Electrolysis,” International Journal of Hydrogen Energy 49 (2024): 616–624.

[52] D. Pergolesi , E. Fabbri , and E. Traversa , “Chemically Stable Anode‐Supported Solid Oxide Fuel Cells Based on Y‐Doped Barium Zirconate Thin Films Having Improved Performance,” Electrochemistry Communications 12 (2010): 977–980.

[53] C. Duan , J. Tong , M. Shang , et al., “Readily Processed Protonic Ceramic Fuel Cells with High Performance at Low Temperatures,” Science 349 (2015): 1321–1326. 26217064 10.1126/science.aab3987

[54] Z. Yue , M. Guo , Z. Chen , et al., “Evolution of PrBa_0.5_Sr_0.5_Co_1.5_Fe_0.5_O_5+δ_ cathode/BaZr_0.1_Ce_0.7_Y_0.1_Yb_0.1_O_3−δ_ Electrolyte Interface of Protonic Ceramic Fuel Cells,” International Journal of Hydrogen Energy 190 (2025): 152247.

[55] Y. Chen , Y. Choi , S. Yoo , et al., “A Highly Efficient Multi‐Phase Catalyst Dramatically Enhances the Rate of Oxygen Reduction,” Joule 2 (2018): 938–949.

[56] F. He , D. Song , R. Peng , G. Meng , and S. Yang , “Electrode Performance and Analysis of Reversible Solid Oxide Fuel Cells with Proton Conducting Electrolyte of BaCe_0.5_Zr_0.3_Y_0.2_O_3−δ_ ,” Journal of Power Sources 195 (2010): 3359–3364.

[57] H. Tao , W. Tang , X. Zhao , and L. Zhu , “Enhanced Stability of Ammonia‐Fed Protonic Ceramic Fuel Cells via Infiltration of Co–Fe Catalyst,” International Journal of Hydrogen Energy 121 (2025): 70–78.

[58] Y. Huan , F. Zhu , J. Zeng , et al., “Effect of Synthesis Methods on Microstructure and Electrochemical Performance of PrBa_0.5_Sr_0·5_Co_1·5_Fe_0·5_O_5+_ * _δ_ * Cathodes for Protonic Ceramic Fuel Cells,” International Journal of Hydrogen Energy 160 (2025): 150645.

[59] H. Zhang , K. Xu , F. He , et al., “Surface Regulating of a Double‐Perovskite Electrode for Protonic Ceramic Fuel Cells to Enhance Oxygen Reduction Activity and Contaminants Poisoning Tolerance,” Advanced Energy Materials 12 (2022): 2200761.

[60] H. S. Park , H. J. Jeong , K.‐H. Kim , et al., “High‐Performance Proton Ceramic Fuel Cells Using a Perovskite Oxide Cathode Surface Decorated with CoO_x_ Nanoparticles,” Applied Surface Science 612 (2023): 155812.

[61] H. Lee , S. Lee , T. Lee , S. Park , and D. Shin , “Long Term Stability of Porosity Gradient Composite Cathode Controlled by Electro‐static Slurry Spray Deposition,” International Journal of Hydrogen Energy 42 (2017): 3748–3752.

[62] X. Chen , H. Zhang , Y. Li , et al., “Fabrication and Performance of Anode‐Supported Proton Conducting Solid Oxide Fuel Cells Based on BaZr_0.1_Ce_0.7_Y_0.1_Yb_0.1_O_3‐_ * _δ_ * Electrolyte by Multi‐Layer Aqueous‐Based Co‐Tape Casting,” Journal of Power Sources 506 (2021): 229922.

[63] M. Marrony and J. Dailly , “Advanced Proton Conducting Ceramic Cell as Energy Storage Device,” Journal of The Electrochemical Society 164 (2017): F988–F994.

