# Supplementary material for: Fabrication of BaZr0.1Ce0.7Y0.1Yb0.1O3‐δ Skeleton Structure for the Development of High‐Performance Nanocomposite Cathodes for Protonic Ceramic Fuel Cells by Nitrate Impregnation
Source: Small. 2026 Jul 25;22(52):e74677. doi: 10.1002/smll.74677 (PMC13580273; doi:10.1002/smll.74677)
Supplement: Supplementary file 1 [file SMLL-22-e74677-s002.docx]

Supporting Information

**Fabrication of BaZr_0.1_Ce_0.7_Y_0.1_Yb_0.1_O_3-δ_ skeleton structure for the development of high-performance nanocomposite cathodes for protonic ceramic fuel cells by nitrate impregnation**

*Jialin Li, Zhongwei Yue, Zhishan Li^*^, Yu Shen^*^, Fang Wang^*^, and San Ping Jiang^*^*

**Table of Contents**

**Supplementary Figures**

**Figure S1** Diameter size distributions of BZCYYb1711 skeleton.

**Figure S2** Diameter size distributions of BZCYYb1711 electrolyte surface.

**Figure S3** EDX results of pristine BZCYYb1711 electrolyte surface.

**Figure S4** EDX results of PBSCF-1711 skeleton.

**Figure S5** EIS spectra of symmetric cells.

**Figure S6** Constant current polarization curves in three-electrode-cells.

**Figure S7** I-V, I-P curves and EIS spectra.

**Figure S8** SEM results of symmetric cells.

**Figure S9** SEM results of single cells.

**Figure S10** EIS spectra of symmetric cell and DRT results.

**Figure S11** BET results of BZCYYb1711 .

**Supplementary Tables**

**Table S1** Lattice constant of LSM, LSCF and PBSCF powders.

**Table S2** TECs of BZCYYb1711, LSM, LSCF and PBSCF.

**Table S3** Voltage decay rates of air electrodes in PCFCs.

**List of Figures:**

**Figure S1.** Cross-section of BZCYYb1711 electrolyte with BZCYYb1711 skeleton sintered at (a) 900 °C, (b) 1000 °C, (c) 1100 °C, (d) 1200 °C for 2 h and the respective diameter size distributions measured by ImageJ (e).

**Figure S2.** Surface of pristine BZCYYb1711 electrolyte (a), with BZCYYb1711 skeleton sintered at (b) 900 °C, (c) 1000 °C and (d) 1200 °C for 2 h and the respective diameter size distributions measured by ImageJ. The BZCYYb1711 skeleton was removed by sticking paper method.

**Figure S3.** Surface SEM images and the corresponding EDX scan analysis of pristine BZCYYb1711 electrolyte (a) and 1711 after removal of skeleton(b). EDX scan results and source data of 1-4 points on surface of PBSCF-1711 after removal of skeleton (c and d).

**Figure S4.** Cross-section SEM images and the corresponding EDX scan analysis of PBSCF-1711 skeleton.

**Figure S5.** (a) EIS results of symmetric cells with 1711 (a), LSM-1711 (b), LSCF-1711 (c) and PBSCF-1711 (d) cathodes in wet air at 550-700 °C.

**Figure S6.** Durability of (a) 1711, (b) LSM-1711, (c) LSCF-1711 and (d) PBSCF-1711 cathodes at 700 °C in wet air under a current density of 0.5 A cm^-2^.

**Figure S7.** (a) Typical current-voltage-power (I-V-P) curves of single cell with 1711 cathode. (b) EIS results of the single cell with 1711 cathode measured at 550-700 °C. (c) EIS results of the single cell with PBSCF-1711-5 cathode measured at 550, 600 and 700 °C.

**Figure S8.** (a)Cross-sectional SEM images of symmetric cell with BZCYYb1711 skeleton sintered at (a)900°C, (b)1000°C (c)1100°C, and (d) 1200°C.

**Figure S9.** Cross-sectional SEM images of single cell with PBSCF-1711-5 electrode (a) before test, (b) after 300 h of operation in FC mode and (c) after 100 h of reversible cycling operation.

**Figure S10.** (a) EIS results of symmetric cells with LSCF-1711 cathode measured under different p_H2O_ at 500 °C. (b) DRT curves.

**Figure S11.** BET adsorption isotherm plot of BZCYYb1711.

**List of Tables:**

**Table S1** Lattice parameters of LSM, LSCF and PBSCF powders from Rietveld refinements in **Figure. 1**.

| Sample | Lattice parameters (Å) | | | Cell column (Å^3^) |
| --- | --- | --- | --- | --- |
|  | a | b | c |  |
| LSM | 5.516 | 5.516 | 13.356 | 351.886 |
| LSCF | 5.522 | 5.483 | 7.797 | 236.078 |
| PBSCF | 5.493 | 7.775 | 5.499 | 234.845 |

**Table S2** List of liner thermal expansion coefficients (TECs) of BZCYYb1711, LSM, LSCF and PBSCF.

| Sample | TEC**/**K^-1^ | Temperature range (°C) | Atmosphere | Ref. |
| --- | --- | --- | --- | --- |
| BZCYYb1711 | 9.59×10^-6^ | 30-700 | Air | This work |
| BZCYYb1711 | 9.7×10^-6^ | 0-200 | Dry N_2_ | ^1^ |
| BZCYYb1711 | 9.7×10^-6^ | 0-200 | Wet N_2_ | ^1^ |
| LSM | 13.35×10^-6^ | 30-700 | Air | This work |
| LSM | 11.3×10^-6^ | 600-1100 | Air | ^2^ |
| LSM | 12.03×10^-6^ | 30-800 | Air | ^3^ |
| LSCF | 11.48×10^-6^ | 30-700 | Air | This work |
| LSCF | 13.6×10^-6^ | 20-800 | Air | ^4^ |
| LSCF | 17×10^-6^ | 20-1000 | Air | ^5^ |
| PBSCF | 16.66×10^-6^ | 30-700 | Air | This work |
| PBSCF | 21.3×10^-6^ | 20-900 | Air | ^6^ |
| PBSCF | 24.3×10^-6^ | 50-850 | Air | ^7^ |

**Table S3** Voltage decay rates of air electrodes in PCFCs during long-term current polarization.

| Air electrode | Operating time (h) | Atmosphere | Voltage decay rate (h^-1^) | Ref. |
| --- | --- | --- | --- | --- |
| PBSCF-1711-5 | 734 | Ambient air | 0.013 % | This work |
| PBSCF | 100 | Ambient air | 0.06 % | ^8^ |
| PBSCF | 100 | Dry air | 0.05 % | ^9^ |
| PBSCF | 100 | Dry air | 0.092 % | ^10^ |
| PNC-LSCF | 200 | - | 0.039 % | ^11^ |
| LSCF | 100 | Air | 0.025 % | ^12^ |
| LSCF | 100 | Air | 0.056 % | ^13^ |

**References**

(1) Nomura, K.; Shimada, H.; Yamaguchi, Y.; Shin, W.; Okuyama, Y.; Mizutani, Y. Phase Transitions, Thermal Expansions, Chemical Expansions, and CO*_2_* Resistances of Ba(Ce*_0.8-x_*Zr*_x_*Y*_0.1_*Yb*_0.1_*)O*_3-δ_* (x = 0.1, 0.4) Perovskite-Type Proton Conductors. *Journal of The Electrochemical Society* **2022**, *169* (2), 024516. DOI: <https://doi.org/10.1149/1945-7111/ac5480>.

(2) Mori, M.; Hiei, Y.; Sammes, N. M.; Tompsett, G. A. Thermal‐Expansion Behaviors and Mechanisms for Ca‐ or Sr‐Doped Lanthanum Manganite Perovskites under Oxidizing Atmospheres. *Journal of The Electrochemical Society* **2000**, *147* (4), 1295. DOI: <https://doi.org/10.1149/1.1393353>.

(3) Xu, Y.; Guo, X.; Lin, C.; Jiang, C.; Zheng, W.; Chang, C.; Zeng, Y. Thermal Properties and Microstructures Analysis of YSZ and YSZ-Al*_2_*O*_3_* Thermal Barrier Coatings. *Journal of Thermal Spray Technology* **2020**, *29* (4), 574-581. DOI: <https://doi.org/10.1007/s11666-020-00996-w>.

(4) Sun, Y.; Jin, D.; Zhang, X.; Shao, Q.; Guan, C.; Li, R.; Cheng, F.; Lin, X.; Xiao, G.; Wang, J. Controllable Technology for Thermal Expansion Coefficient of Commercial Materials for Solid Oxide Electrolytic Cells. *Materials (Basel)* **2024**, *17* (5). DOI: <https://doi.org/10.3390/ma17051216> From NLM.

(5) Vibhu, V.; Yildiz, S.; Vinke, I. C.; Eichel, R. A.; Bassat, J. M.; de Haart, L. G. J. High Performance LSC Infiltrated LSCF Oxygen Electrode for High Temperature Steam Electrolysis Application. *Journal of The Electrochemical Society* **2019**, *166* (2), F102. DOI: <https://doi.org/10.1149/2.0741902jes>.

(6) Jiang, L.; Wei, T.; Zeng, R.; Zhang, W.-X.; Huang, Y.-H. Thermal and electrochemical properties of PrBa*_0.5_*Sr*_0.5_*Co*_2−x_*Fe*_x_*O*_5+δ_* (x = 0.5, 1.0, 1.5) cathode materials for solid-oxide fuel cells. *J. Power Sources* **2013**, *232*, 279-285. DOI: <https://doi.org/10.1016/j.jpowsour.2013.01.064>.

(7) Hung, I. M.; Mohanty, D.; Lin, Y.-R.; Lee, S.-W.; Tseng, C.-J.; Chen, Y.-W. Synthesis and Electrochemical Performance of PrBa*_0.5_*Sr*_0.5_*Co*_1.5_*Fe*_0.5_*O*_5+δ_* double perovskite cathode material for Solid Oxide Fuel Cell. *International Journal of Hydrogen Energy* **2025**, *145*, 371-379. DOI: <https://doi.org/10.1016/j.ijhydene.2025.06.124>.

(8) Huan, Y.; Zhu, F.; Zeng, J.; Zhang, L.; Jian, C.; Huang, Y.; Chen, Y. Effect of synthesis methods on microstructure and electrochemical performance of PrBa*_0.5_*Sr*_0·5_*Co*_1·5_*Fe*_0·5_*O*_5+δ_* cathodes for protonic ceramic fuel cells. *International Journal of Hydrogen Energy* **2025**, *160*, 150645. DOI: <https://doi.org/10.1016/j.ijhydene.2025.150645>.

(9) Zhang, H.; Xu, K.; He, F.; Zhou, Y.; Sasaki, K.; Zhao, B.; Choi, Y.; Liu, M.; Chen, Y. Surface Regulating of a Double-Perovskite Electrode for Protonic Ceramic Fuel Cells to Enhance Oxygen Reduction Activity and Contaminants Poisoning Tolerance. *Advanced Energy Materials* **2022**, *12* (26), 2200761. DOI: <https://doi.org/10.1002/aenm.202200761> (acccessed 2026/01/25).

(10) Park, H. S.; Jeong, H. J.; Kim, K.-h.; Chang, W.; Kim, Y. S.; Choi, Y. S.; Shim, J. H. High-performance proton ceramic fuel cells using a perovskite oxide cathode surface decorated with CoO*_x_* nanoparticles. *Applied Surface Science* **2023**, *612*, 155812. DOI: <https://doi.org/10.1016/j.apsusc.2022.155812>.

(11) Yao, P.; Zhang, J.; Qiu, Q.; Zhao, Y.; Yu, F.; Li, Y. Enhancing Oxygen Reduction Kinetics and Proton Transfer of La*_0.6_*Sr*_0.4_*Co*_0.2_*Fe*_0.8_*O*_3−δ_* Cathode through Pr*_2_*Ni*_0.5_*Co*_0.5_*O*_4−δ_* Impregnation for Protonic Ceramic Fuel Cells. *Advanced Energy Materials* **2025**, *15* (9), 2403335. DOI: <https://doi.org/10.1002/aenm.202403335> (acccessed 2025/08/27).

(12) Lee, H.; Lee, S.; Lee, T.; Park, S.; Shin, D. Long term stability of porosity gradient composite cathode controlled by electro-static slurry spray deposition. *International Journal of Hydrogen Energy* **2017**, *42* (6), 3748-3752. DOI: <https://doi.org/10.1016/j.ijhydene.2016.09.077>.

(13) Chen, X.; Zhang, H.; Li, Y.; Xing, J.; Zhang, Z.; Ding, X.; Zhang, B.; Zhou, J.; Wang, S. Fabrication and performance of anode-supported proton conducting solid oxide fuel cells based on BaZr*_0.1_*Ce*_0.7_*Y*_0.1_*Yb*_0.1_*O*_3-δ_* electrolyte by multi-layer aqueous-based co-tape casting. *J. Power Sources* **2021**, *506*, 229922. DOI: <https://doi.org/10.1016/j.jpowsour.2021.229922>.
